# IL-4/IL-4R axis signaling drives resistance to immunotherapy by inducing the upregulation of Fcγ receptor IIB in M2 macrophages

**DOI:** 10.1038/s41419-024-06875-4

**Published:** 2024-07-13

**Authors:** Jiayu Zhang, Yu Dong, Shan Yu, Keshu Hu, Lingyun Zhang, Min Xiong, Mengling Liu, Xun Sun, Suyao Li, Yitao Yuan, Chi Zhang, Mengxuan Zhu, Yichou Wei, Yanjing Zhu, Yiyi Yu, Pengfei Zhang, Tianshu Liu

**Affiliations:** 1 Department of Medical Oncology, Zhongshan Hospital, Fudan University, Shanghai, China; 2 Cancer Center, Zhongshan Hospital, Fudan University, Shanghai, China; 3 Tianjin Medical University Cancer Institute and Hospital, National Clinical Research Center for Cancer, Key Laboratory of Cancer Prevention and Therapy, Tianjin’s Clinical Research Center for Cancer, Tianjin, 300060 China; 4 Department of Breast Surgery, Key Laboratory of Breast Cancer in Shanghai, Fudan University Shanghai Cancer Center, Shanghai, China

**Keywords:** Gastric cancer, Predictive markers

## Abstract

In recent years, immunotherapy, particularly PD-1 antibodies, have significantly enhanced the outcome of gastric cancer patients. Despite these advances, some patients do not respond well to treatment, highlighting the need to understand resistance mechanisms and develop predictive markers of treatment effectiveness. This study retrospectively analyzed data from 106 patients with stage IV gastric cancer who were treated with first-line immunotherapy in combination with chemotherapy. By comparing plasma cytokine levels between patients resistant and sensitive to PD-1 antibody therapy, the researchers identified elevated IL-4 expression in the resistant patients. Mechanical investigations revealed that IL-4 induces metabolic changes in macrophages that activate the PI3K/AKT/mTOR pathway. This alteration promotes ATP production, enhances glycolysis, increases lactic acid production, and upregulates FcγRIIB expression in macrophages. Ultimately, these changes lead to CD8+ T cell dysfunction and resistance to PD-1 antibody therapy in gastric cancer. These findings highlight the role of IL-4-induced macrophage polarization and metabolic reprogramming in immune resistance and verify IL-4 as potential targets for improving treatment outcomes in gastric cancer patients.

## Background

According to the global cancer statistics in 2020, gastric cancer ranks fifth in morbidity and fourth in mortality worldwide [1]. Due to the atypical symptoms of early gastric cancer and the insufficient popularization of screening for gastric cancer, it is reported that one-third of patients have developed distant metastases at the time of initial diagnosis [2], which means that such patients cannot get radical surgery in the short term, and can only take systemic antitumor therapy. In recent years, immunotherapy represented by immune checkpoint inhibitors, especially PD-1 antibody, has effectively improved the prognosis of gastric cancer patients. Since the activation and killing effects of the immune system are suppressed in the tumor microenvironment, restoring the function of CD8^+^T cells through immunotherapy is currently the focus of research in the field of gastric cancer. With the continuous development of tumor immunology research, PD-1, CTLA-4 and PD-L1 have all become relatively mature immunotherapy targets, among which PD-1 antibody is the most commonly used in the immunotherapy of gastric cancer.

PD-1 is one of the known immune checkpoints, mainly expressed on the surface of activated T cells. PD-1 and its ligands PD-L1 (CD274) or PD-L2 (CD273) play a key role in the immune escape of tumor cells, which can significantly inhibit the function of T cells, while tumors can resist the attack of the immune system through high expression of PD-L1/PD-L2. By blocking the interaction between PD-1 and its ligand, immune system can normally recognize and kill tumors, so as to enhance the anti-tumor activity, which is the core of immunotherapy.

Currently, multiple clinical studies have shown that immune checkpoints blocking treatment can effectively enhance anti-tumor activity and improve prognosis in gastric cancer patients. ORIENT-16 study confirmed that first-line immunotherapy combined with chemotherapy could significantly improve prognosis among the whole population with advanced gastric cancer, extending the median OS by 2.9 months, increasing the objective response rate (ORR) from 48.4% to 58.2% and significantly improving the duration of remission (DOR) compared with the chemotherapy group [3]. CheckMate 649 study also confirmed that first-line chemotherapy combined with nivolumab could also significantly benefit the OS or ORR in patients with advanced gastric or gastroesophageal cancer [4]. The results of KEYNOTE-811 clinical study proved that the combination of pembrolizumab, chemotherapy and HER2-targeted therapy could significantly inhibit tumor and improve patients’ objective response rate (ORR), which reached 74.4% [5]. These results suggest that immunotherapy will bring a great change in the treatment of gastric cancer patients, and the treatment of advanced gastric cancer will change from chemotherapy and targeted therapy to combined immunotherapy.

At present, biomarkers related to immunotherapy efficacy can be divided into the following categories: (1) Surface markers, including PD-L1 and some other inhibitory receptors [6]. (2) Gene biomarkers, such as tumor mutational burden (TMB), microsatellite instability (MSI) [7]. (3) Circulating tumor DNA (ctDNA) [8]. Some of these biomarkers have been confirmed by clinical studies and have been widely used clinically. However, although these biomarkers can guide the treatment of gastric cancer patients, the efficacy of immunotherapy in some patients is still unsatisfactory. Therefore, it is necessary to find the key factors affecting the effect of immunotherapy in gastric cancer, in order to promote the efficacy of immunotherapy among gastric cancer patients. At present, we still know little about the mechanism of anti-tumor immune response in gastric cancer. Therefore, it has important clinical guiding significance to fully explore the molecular mechanism of gastric cancer’s tolerance to immunotherapy and develop new predictors of immunotherapy efficacy.

## Results

### IL-4 is a crucial interleukin associated with anti-PD1 tolerance in patients with gastric cancer

**Fig. 1:**
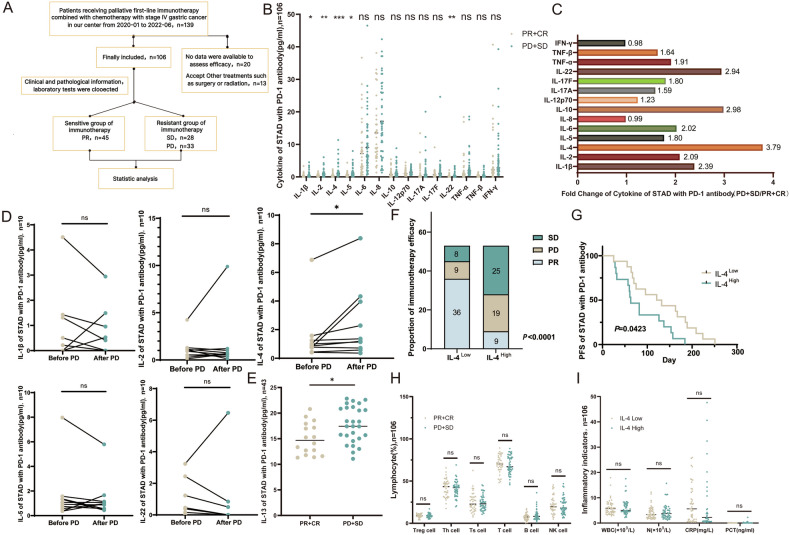
Cytokine IL-4 is associated with immunotherapy resistance in gastric cancer. **A** Clinical cohort collection flow chart. **B** Baseline plasma cytokines in immunotherapy patients with gastric cancer. **C** Fold Change of baseline plasma cytokines in immunotherapy patients with gastric cancer. **D** Plasma cytokines after drug resistance in immunotherapy patients with gastric cancer. **E** Plasma IL-13 levels in immunotherapy patients with gastric cancer. **F** IL-4 levels and immunotherapy efficacy in patients with gastric cancer. **G** Kaplan–Meier analysis of IL-4 levels and immunotherapy PFS in patients with gastric cancer. **H** Proportion of blood lymphocytes in immunotherapy patients with gastric cancer. **I** IL-4 levels and inflammatory markers in immunotherapy patients with gastric cancer.

Previous studies discovered that interleukins expression is associated with immune evasion of a subset of cancers, including gastric cancer [9, 10]. We performed cytokine flow cytometry (include 14 cytokines) to analyze baseline cytokine levels from patients with primary stage IV gastric cancer before received anti-PD1 therapy combined with chemotherapy. The treatment efficacy was monitored by enhanced CT every 3 therapy cycles and assessed according to RECIST 1.1 criteria (Fig. 1A). A total of 106 patients were recruited, and the objective response (ORR) and disease control rates (DCR) were 42% and 69%. Thirty three progressive disease (PD), 28 stable disease (SD), and 45 partial response (PR) were in above patients. We took PR patients as the immunotherapy-sensitive group, SD and PD patients as the immunotherapy-resistant group. Clinical data analysis showed that there were no significant differences between the two groups in terms of gender, mismatch repair (MMR), lauren type, primary site and differentiation degree (Table 1). Among these 14 cytokines, the expression of IL-4 was the most significantly upregulated in gastric cancer patient resistance to anti-PD1 therapy compared to the sensitive group (Fig. 1B). At the same time, in order to further explain the driving effect of IL-4 on immunotherapy resistance of gastric cancer, we further analyzed the Fold Change of cytokine levels in the two groups of patients, and found that the increase of IL-4 was the most significant (PD + SD/PR + CR, fold change = 3.79) (Fig. 1C). Furthermore, patients with secondary resistance are accompanied by elevated IL-4 expression (Fig. 1D), therefore, we conclude that high levels of IL-4 lead to gastric cancer anti-PD-1 immunotherapy resistance. In addition, since IL-13 has generally been shown to have a synergistic effect with IL-4, especially in regulating the function of macrophages, we used ELISA to detect the plasma IL-13 level in the two groups of patients who took blood samples and found that the plasma IL-13 level was increased in the patients in the immunotherapy-resistant group. We hypothesize that IL-13 is synergistic with IL-4 in inducing immunotherapy resistance on the one hand, and that it is the result of cytokine storms induced by tumor progression of resistance on the other (Fig. 1E).

**Table 1:** Gastric cancer immunotherapy cohort baseline data table.

Clinical information (*n* = 106)		Responsed	Non-responsed	*P*
		PR + CR	SD + PD	
		(*n* = 45)	(*n* = 61)	
Age		66 (37–79)	57 (31–79)	0.001
Gender	Male	33 (73.3%)	36 (59%)	0.1264
	Female	12 (26.7%)	25 (41%)	
PD-L1 of tumor	Negative	20 (44.4%)	38 (62.3%)	0.0699
	Positive	12 (26.6%)	9 (14.7%)	
	Unknown	13 (29%)	14 (23%)	
PD-L1 of combination	Negative	11 (24.4%)	23 (37.7%)	0.23
	Positive	21 (46.7%)	25 (41%)	
	Unknown	13 (28.9%)	13 (21.3%)	
MMR	dMMR	4 (8.9%)	4 (6.6%)	0.8028
	pMMR	37 (82.2%)	53 (86.9%)	
	Unknown	4 (8.9%)	4 (6.6%)	
Location	Gastric body	21 (46.7%)	32 (52.5%)	0.3706
	Gastric antrum	12 (26.7%)	20 (32.8%)	
	Cardia	10 (22.2%)	6 (9.8%)	
	Unknown	2 (4.4%)	3 (4.9%)	
Differentiation	Low	19 (42.2%)	28 (45.9%)	0.9489
	Medium low	11 (24.4%)	13 (21.3%)	
	Moderate	10 (22.2%)	12 (19.7%)	
	Unknown	5 (11.1%)	8 (13.1)	
Lauren type	Intestinal type	18 (40%)	18 (29.5%)	0.2728
	Diffuse type	7 (15.6%)	14 (23%)	
	Mixed type	8 (17.8%)	18 (19.5%)	
	Unknown	12 (26.7%)	11 (18%)	

We divided 106 patients into two groups according to plasma baseline IL-4 levels, and compared the efficacy of immunotherapy. We found that the proportion of drug-resistant patients in the L-4^high^ group was higher, and the proportion of sensitive patients in the l IL-4^low^ group was higher, with a higher objective remission rate (67.9% vs 17.0%) (Fig. 1F). In addition, Kaplan–Meier survival analysis indicated that the PFS of the IL-4^high^ group was much lower than that for the IL-4^low^ group (Fig. 1G). The median PFS was 63 days in the IL-4^high^ group and **127** days in the IL-4^low^ group. We also counted the cellular immune indicators in the baseline laboratory examination of the above 106 patients, analyzed the proportion of lymphoid cells in the blood of immunotherapy patients, and found that there was no significant difference in T cells, B cells, NK cells among two groups of patients (Fig. 1H). In order to avoid the increase of cytokine levels caused by the patients’ own inflammatory response, we calculated the inflammatory indicators of all patients and divided them into two groups according to the plasma baseline IL-4 level, which was no significant difference (Fig. 1I).

In addition, to further confirm the role of IL-4/IL-4R axis in gastric cancer, analysis of TCGA data showed that high expression of IL-4R was correlated with poor OS and PFS in patients with gastric cancer (Supplementary Fig. 1A, B). At the same time, IL-4 and IL-4R were highly expressed in tumor tissues (Supplementary Fig. 1C, D), while in the public database, MSIscore and TMB in gastric cancer tissues were decreased with the increase of IL-4 and IL-4R expression (Supplementary Fig. 1E–H).

### IL-4 inhibit PD-1 antibody effect in gastric cancer in vivo

**Fig. 2:**
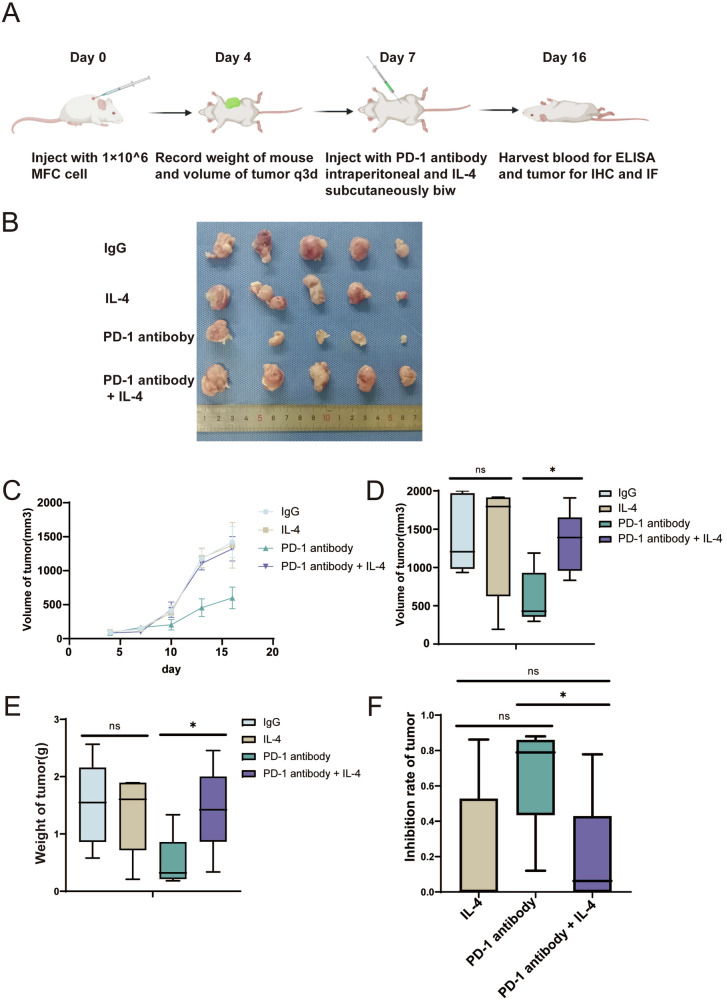
IL-4 inhibit PD-1 antibody efficacy in gastric cancer in vivo. **A** Animal model construction. **B** MFC cells were used to construct xenograft tumors in 615 mice. **C**, **D** Tumor size was measured after xenograft tumor was formed. **E**, **F** Tumor weighing and inhibition rate calculation after xenograft tumor was formed.

To further determine the effects of IL-4 on anti-PD-1 therapy resistance, we examined the anti-tumor effects of the PD-1 antibody in xenograft 615 mice that received MFC cells (Fig. 2A). Compared to that of the PD-1 antibody group, the tumor growth in the IL-4 recipient xenograft mice showed an obvious phenotype of resistance to anti-PD-1 therapy (Fig. 2B–F).

### IL-4 is associated with macrophage M2 polarization in gastric cancer tissues

Significant macrophage infiltration has been demonstrated in mouse models of gastric cancer, and these macrophages are recruited by chemokines and cytokines in the tumor microenvironment, and depletion of macrophages can inhibit tumor growth in vivo [11]. Therefore, cibersort algorithm was used to analyze the RNA-seq data of gastric cancer in TCGA, and it was found that compared with normal tissues, macrophage infiltration in tumors was significantly increased, accompanied by increased Treg cell infiltration and decreased plasma cell infiltration, which confirmed that the immune microenvironment of gastric cancer was in a state of immunosuppression (Supplementary Fig. 2A, B). We analyzed the relationship between immune cell infiltration and patient prognosis in TCGA gastric cancer data, and found that macrophages were significantly negatively correlated with patient survival (Supplementary Fig. 2C).

In addition, xCELL immunoinfiltration analysis was performed on the gastric cancer RNA-seq data of TCGA, and it was found that M2 macrophage infiltration increased and Th1 and Th2 cell infiltration decreased with the activation of IL-4/IL-4R axis (Supplementary Fig. 3A). The analysis of the gastric cancer sequencing data of TCGA showed that the expression of IL-4R was strongly correlated with the infiltration of macrophages (Supplementary Fig. 3B).

**Fig. 3:**
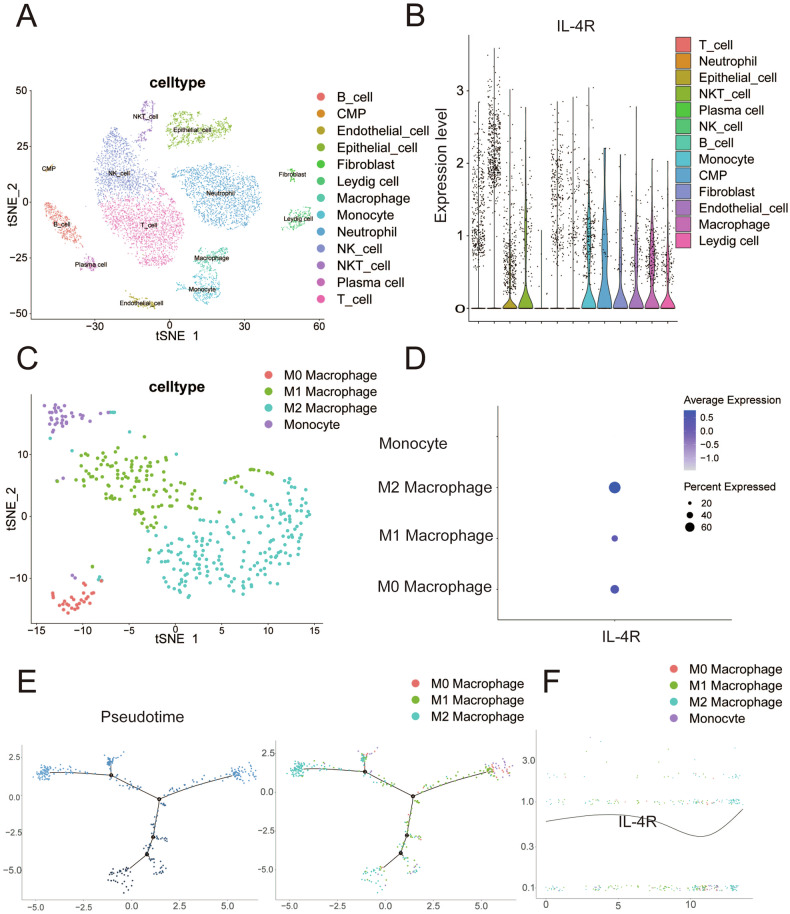
IL-4R and gastric cancer macrophages in single cell sequencing. **A** TSNE cluster map of gastric cancer single cell sequencing data. **B** Expression of IL-4R in each subgroup of gastric cancer single cell sequencing data, **C** TSNE cluster of macrophage subsets from gastric cancer single cell sequencing data. **D** Expression of IL-4R in macrophage subsets of gastric cancer single cell sequencing data. **E** Pseudo-time series analysis of macrophage subsets of gastric cancer single cell sequencing data. **F** Expression of IL-4R in macrophage pseudo-time series analysis.

We analyzed the single-cell sequencing data GSE163558 and used the expression level of IL-4R to represent the activation level of IL-4/IL-4R axis on each cell [12]. We found that among the 13 cell subsets isolated from gastric cancer tissues, IL-4R was mainly expressed in macrophages, monocytes, stromal cells and epithelial cells (Fig. 3, B). After subpopulation analysis of macrophages, it was found that IL-4R was mainly highly expressed in M2 macrophages (Fig. 3C, D). We conducted a quasi-temporal analysis of the subsets of macrophages and found that M1 and M2 macrophages were in the late stage of macrophage development (Fig. 3E), and with the increasing expression of IL-4R, macrophages gradually transformed into M2 macrophages (Fig. 3F).

In addition, we detected the expression of downstream molecules of IL-4/IL-4R axis, such as PI3K, AKT1, AKT2 and STATA6 in GSE163558. We found that they were expressed in macrophages (Supplementary Fig. 4A–D). At the same time, the subsets of macrophages were analyzed, and it was found that the expressions of PI3K, AKT1, AKT2 and STATA6 were overexpressed in M2 macrophages (Supplementary Fig. 4E–H), which also confirmed our opinion of IL-4/IL-4R axis activation in M2 macrophages.

### IL-4/IL-4R axis is crucial for monocytes metabolic reprogramming and differentiates towards M2 macrophages

Previous study has reported that IL-4-mediated enhanced aerobic glycolysis is essential for M2 macrophage activation [13]. THP1 cells and U937 cells were stimulated by PMA combined with or without IL-4 for 48 h (Supplementary Fig. 5A). The results of qRT-PCR showed that the expression of M2 macrophage markers (CD206, CD163, Arg1) was up-regulated and the expression of M1 macrophage markers (INOS, IL-1β) was downregulated in macrophages co-cultured with IL-4 (Supplementary Fig. 5B, C). Furthermore, the flow cytometry analysis demonstrated that CD206, a specific marker for M2 macrophages was significantly upregulated in THP1 cells and U937 cells (Supplementary Fig. 5E, F). In addition, immunofluorescence of mouse subcutaneous cancer specimens showed that macrophage marker F4/80 was not significantly different among the groups, while M2 macrophage marker CD206 was upregulated in the two groups injected with IL-4 (Supplementary Fig. 5G, H).

**Fig. 4:**
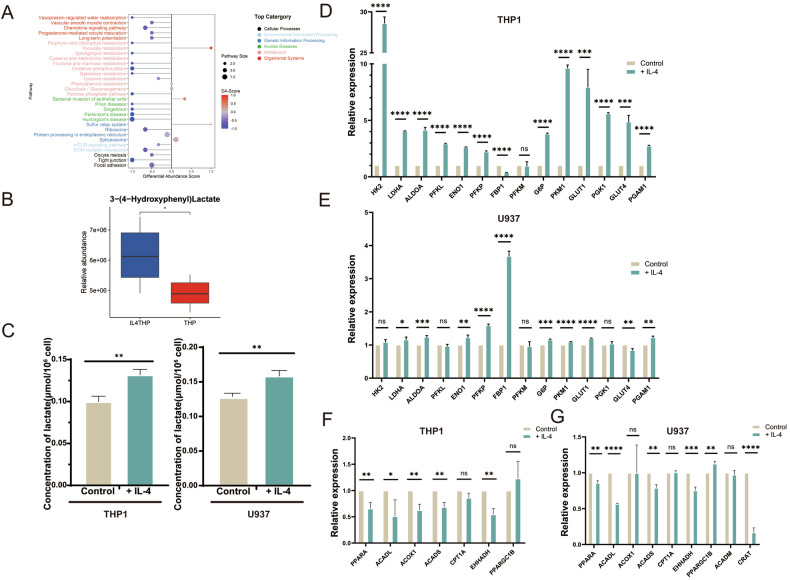
IL-4 mediates metabolic reprogramming of M2 macrophages. **A** Overall differential metabolite enrichment analysis. **B** The concentration of lactic acid was detected by mass spectrometry. **C** The concentration of lactic acid was detected by colorimetry. **D** The key gene expression of glycolysis in THP1 after IL-4 stimulation was detected by qRT-PCR. **E** The key gene expression of glycolysis in U937 after IL-4 stimulation was detected by qRT-PCR. **F** The key gene expression of oxidative phosphorylation in THP1 after IL-4 stimulation was detected by qRT-PCR. **G** The key gene expression of oxidative phosphorylation in U937 after IL-4 stimulation was detected by qRT-PCR.

In previous studies, mouse bone marrow-derived macrophages were used for metabolic differential analysis [13]. Non-targeted metabolic sequencing of IL-4-stimulated THP1 showed that oxidative phosphorylation and tricarboxylic acid cycling in macrophages were inhibited after IL-4 stimulation, and pyruvate metabolism was enhanced (Fig. 4A). Although there were no significant differences in glycolysis, the production of lactate increased after IL-4 stimulation (Fig. 4B). In addition, some of the upregulated differential metabolites were also fatty acid metabolites, which was also consistent with the metabolic characteristics of M2 macrophages. We measured the lactic acid content of THP1 and U937 after IL-4 stimulation, and found that the production of lactic acid in macrophages increased significantly after IL-4 stimulation (Fig. 4C). Importantly, our results also demonstrated that the expression of aerobic glycolysis genes LDHA, ALODA, ENO1, PFKP, G6P, PKM1, GLUT1, GLUT4, PGAM1 were significantly increased in the IL-4 stimulated THP1 cells and U937 cells (Fig. 4D, E), while the key genes of oxidative phosphorylation are inhibited (Fig. 4F, G).

### IL-4 mediates metabolic reprogramming of macrophages by upregulating the PI3K-AKT-mTOR signaling pathway

**Fig. 5:**
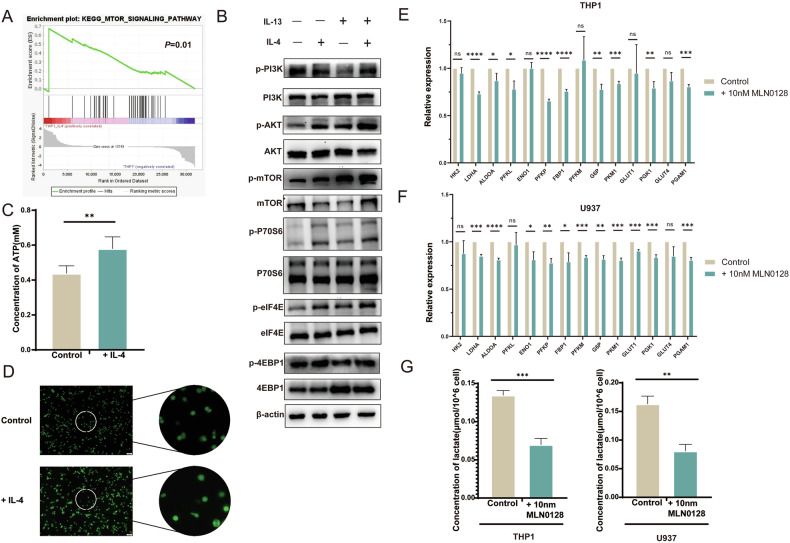
The PI3K/AKT/mTOR signaling pathway mediates metabolic reprogramming of M2 macrophages. **A** Analysis of THP1 RNA-seq GSEA enrichment after IL-4 stimulation. **B** Western blot analysis of PI3K/AKT/mTOR signaling pathway in THP1 after IL-4 stimulation. **C** The concentration of ATP in THP1 after IL-4 stimulation was measured by colorimetry. **D** ROS levels in THP1 after IL-4 stimulation were detected by fluorescent probe. **E** The expression of key genes of glycolysis in THP1 after mTOR inhibitor stimulation was detected by qRT-PCR. **F** The expression of key genes of glycolysis in U937 after mTOR inhibitor stimulation was detected by qRT-PCR. **G** The lactate concentration of M2 macrophages was measured by colorimetry after mTOR inhibitor stimulation.

RNA-seq of THP1 after IL-4 stimulation revealed activation of the mTOR signaling pathway (Fig. 5A). Later, western blot was used for verification, and it was found that the PI3K/AKT/mTOR signaling pathway was up-regulated in macrophages stimulated by IL-4: Phosphorylation levels of PI3K, AKT, and mTOR were increased, and phosphorylation levels of P70S6 and elF4E [14] were also enhanced to varying degrees (Fig. 5B). In addition, we found that macrophages stimulated by IL-4 increased ATP concentration (Fig. 5C) and reactive oxygen species (ROS) levels (Fig. 5D). We treated THP1 and U937-derived M2 macrophages with the mTORC1/C2 inhibitor MLN0128, and found that the expression of key genes in glycolysis was inhibited in macrophages (Fig. 5E, F), while lactate production was inhibited (Fig. 5G). Finally, it was confirmed that the up-regulation of PI3K/AKT/mTOR signaling pathway regulated the metabolic reprogramming of macrophages during the process of IL-4 promoting the polarization of M2 macrophages.

In addition to type I IL-4, the effect of type II IL-4R on IL-4 activation of PI3K-AKT-mTOR signaling pathway was investigated. Since the receptor that binds IL-4 is IL-4Rα, both type I and type II IL-4R contain IL-4rα, but the other subunits are different. Therefore, in a follow-up experiment, we knocked down IL-13Rα1 in type II IL4R. After verifying the knockdown efficiency (Supplementary Fig. 6A), we found that the PI3K-AKT-mTOR signaling pathway activation of THP1 after IL-4 stimulation was not affected (Supplementary Fig. 6B).

### Macrophage mediated immune escape after IL-4 stimulation

**Fig. 6:**
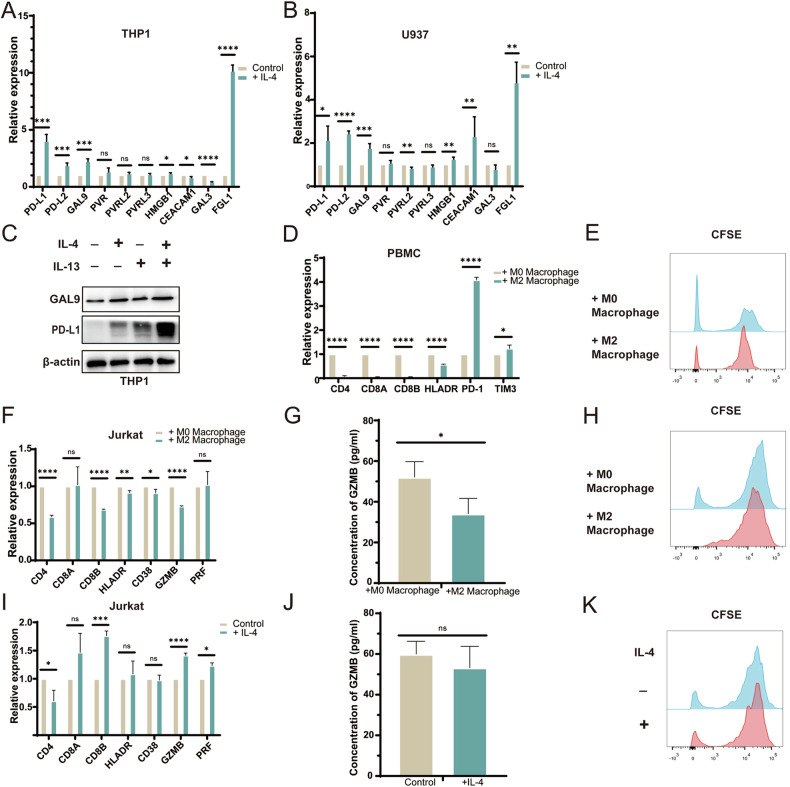
Macrophage mediated immune escape after IL-4 stimulation. **A** The expression of immune checkpoint ligand in THP1 after IL-4 stimulation was detected by qRT-PCR. **B** The expression of immune checkpoint ligand in U937 after IL-4 stimulation was detected by qRT-PCR. **C** Western blot analysis of PD-L1 and GAL9 in THP1 after IL-4 stimulation. **D** Immunosuppression of PBMC derived T cells co-cultured with M2 macrophages was detected by qRT-PCR. **E** Proliferative ability of PBMC-derived T cells co-cultured with M2 macrophages was detected by CFSE experiments. **F** Immunosuppression of Jurkat cells co-cultured with M2 macrophages was detected by qRT-PCR. **G** Immunosuppression of Jurkat cells co-cultured with M2 macrophages was detected by ELISA. **H** Proliferative ability of Jurkat cells co-cultured with M2 macrophages was detected by CFSE experiments. **I** Immunosuppression of Jurkat cells after PMA and IL-4 stimulation was detected by qRT-PCR. **J** Immunosuppression of Jurkat cells after PMA and IL-4 stimulation was detected by ELISA. **K** Proliferative ability of Jurkat cells after PMA and IL-4 stimulation was detected by CFSE experiments.

To further investigate the mechanism through which IL-4 promotes an immune evasion profile in macrophages, we found that IL-4 significantly increased the expression of Immune checkpoint ligands such as PD-L, PD-L2, GAL9, HMGB1, FGL1 on monocytes-derived M2 macrophages (Fig. 6A–C). Previous studies have shown that the benefit of pembrolizumab treatment in gastric cancer patients is related to the infiltration of CD8^+^T cells [15]. Monocyte-derived macrophages stimulated by IL-4 were co-cultured with T cells (PBMC derived T cells from gastric cancer patients, Lymphocytic leukemia cell line Jurkat cells activated by T cell Activator). We found that the expressions of CD4 and CD8 of the two types of T cells were down-regulated, indicating that the proliferation and activation of T cells were inhibited. We analyzed the immune checkpoints on the surface of T cells in PBMC and found that the expression of PD-1 and TIM-3 was up-regulated, indicating that T cell function was inhibited and tended to be disabled and depleted (Fig. 6D). CSFE experiment also confirmed that the proliferation ability of the two types of T cells was impaired after co-culture with M2 macrophages (Fig. 6E, H). Furthermore, in Jurkat cells, after co-culture with M2 macrophages, expression of T-cell killing genes HLADR, CD38, and granzyme B (GZMB) were down-regulated (Fig. 6F), and ELISA results also confirmed that GZMB expression was down-regulated (Fig. 6G). Further, in order to avoid the effect of PMA and IL-4 on T cell function in the co-culture system, no immunosuppression was observed after stimulation of Jurkat cells with PMA and IL-4 alone (Fig. 6I, J). CSFE experiment also confirmed that direct stimulation of IL-4 and PMA did not affect the proliferation ability of T cells (Fig. 6K).

### Lactic acid upregulates the expression of FC-γ Receptor IIB in M2 macrophages

**Fig. 7:**
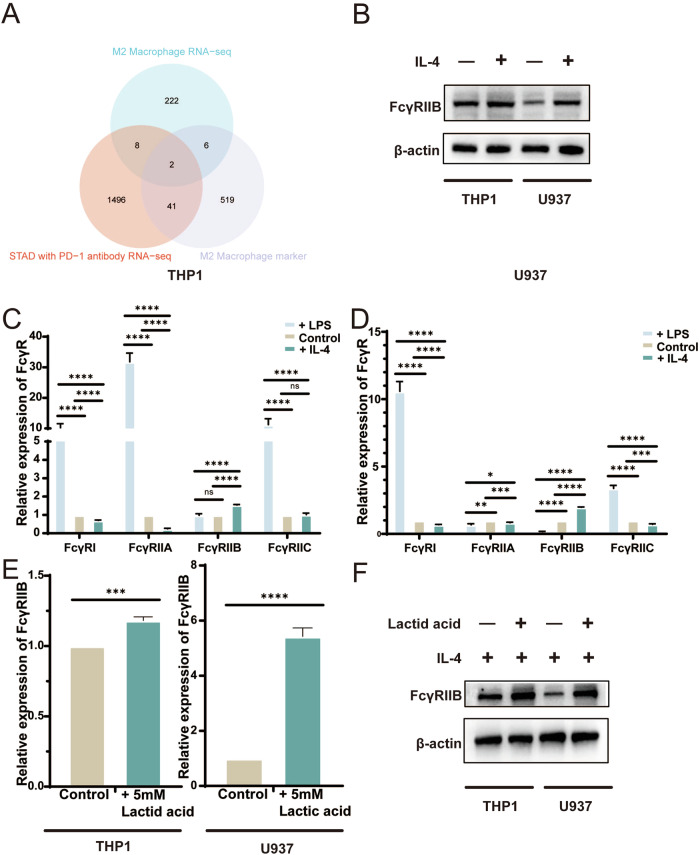
Lactic acid upregulates the expression of FcγRIIB in M2 macrophages. **A** FcγRIIB was chosen based on M2 macrophage RNA-seq, PRJEB25780 and GSE163558. **B** Western blot analysis of FcγR family expression in THP1 cells stimulated by IL-4. **C** The expression of FcγR family in THP1 after IL-4/LPS stimulation was detected by qRT-PCR. **D** The expression of FcγR family in U937 after IL-4/LPS stimulation was detected by qRT-PCR. **E** The expression of FcγRIIB in macrophages after lactic acid stimulation was detected by qRT-PCR. **F** Western blot analysis of FcγRIIB expression in macrophages after lactic acid stimulation.

By analyzing the data of M2 macrophage RNA-seq, PRJEB25780 gastric cancer immunotherapy specimen RNA-seq, and GSE163558 gastric cancer single cell sequence, we identified FcγRIIB, which is highly expressed on the surface of M2 macrophages and is associated with immunotherapy resistance (Fig. 7A). As an immunosuppressive molecule, we confirmed that the expression of FcγRIIB on the surface of macrophages was upregulated after IL-4 stimulation. Interestingly, the expression of other FcγR family molecules that positively regulate immunity was downregulated after IL-4 stimulation, while the opposite trend was found after LPS stimulation of macrophages (Fig. 7B–D). Recently, the end product of glycolysis, lactic acid, was confirmed participated in induces an immunosuppressive characteristic in tumor microenvironment [16, 17]. Therefore, we speculated that lactic acid may be responsible for upregulating the expression levels of FcγRIIB in M2 macrophages. To verify this hypothesis, we analyzed the effect of lactic acid on M2 macrophages. The qRT-PCR and western blot results showed that the expression of FcγRIIB expression was significantly upregulated (Fig. 7E, F).

### Macrophages stimulated by IL-4 upregulates the expression of FcγRIIB by producing acidic microenvironment

**Fig. 8:**
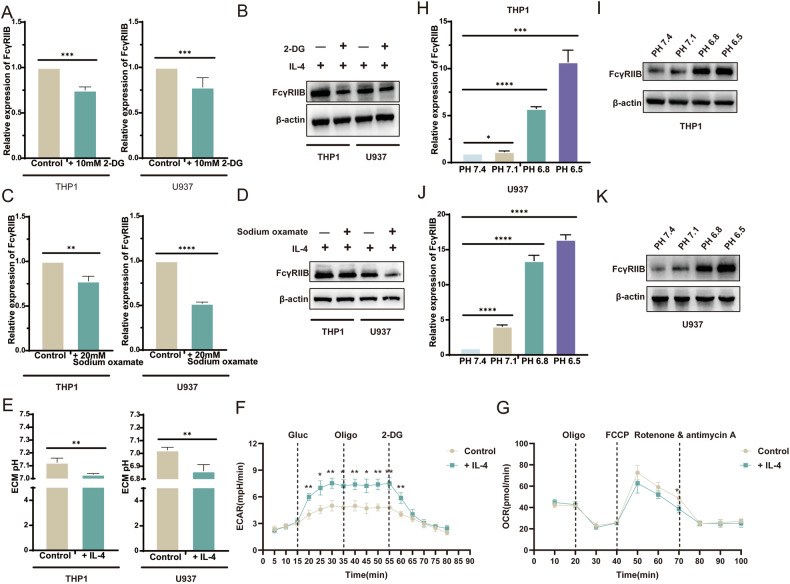
Macrophages stimulated by IL-4 upregulate the expression of FcγRIIB by producing acidic microenvironment. **A** The expression of FcγRIIB in M2 macrophages after 2-DG stimulation was detected by qRT-PCR. **B** Western blot analysis of FcγRIIB expression in M2 macrophages after 2-DG stimulation. **C** The expression of FcγRIIB in M2 macrophages after sodium oxalate stimulation was detected by qRT-PCR. **D** Western blot analysis of FcγRIIB expression in M2 macrophages after sodium oxalate stimulation. **E** Extracellular matrix pH of macrophages after IL-4 stimulation. **F** Detection of THP1 extracellular acidification rate (ECAR) after IL-4 stimulation. **G** Detection of THP1 oxygen consumption rate (OCR) after IL-4 stimulation. **H** The expression of FcγRIIB in THP1 in acidic microenvironment was detected by qRT-PCR. **I** Western blot analysis of FcγRIIB expression in THP1 in acidic microenvironment. **J** The expression of FcγRIIB in U937 in acidic microenvironment was detected by qRT-PCR. **K** Western blot analysis of the expression of FcγRIIB in U937 in acidic microenvironment.

To further investigate whether IL-4-mediated metabolic reprogramming, especially increased lactate production, is associated with the enhancement of FcγRIIB expression on M2 macrophages, we stimulated macrophages with the glycolysis inhibitor 2-deoxyglucose (2-DG). Importantly, our results also showed that 2-Deoxy-D-glucose significantly inhibited FcγRIIB expression in IL-4-stimulated monocytes-derived M2 macrophages (Fig. 8A, B). After the lactate dehydrogenase inhibitor sodium oxalate was used to inhibit the production of lactic acid [18], the expression of FcγRIIB in M2 macrophages was also found to be inhibited (Fig. 8C, D). We hypothesize that lactic acid may affect the level of FcγRIIB in macrophages by promoting the production of acidic microenvironment through release lactic acid into the tumor microenvironment. We measured the extracellular matrix pH of two cell lines by potentiometry using a laboratory precision PH meter. We found that the extracellular matrix pH decreased after IL-4 stimulation (Fig. 8E). The extracellular acidification rate (ECAR) of macrophages stimulated by IL-4 was detected, and it was found that the basal ECARs were higher and exhibited increased glycolytic reserve in monocytes coculture with IL-4 (Fig. 8F). In addition, we also measured the rate of oxygen consumption. We found that THP1 oxygen consumption rate decreased slightly after IL-4 stimulation (Fig. 8G), which is consistent with our results. We attempted to create a tumor acidic microenvironment in vitro. Considering that the tumor acidic microenvironment is mainly maintained by lactic acid produced by cell metabolism, we adjusted pH of the culture solution by gradually adding 1 M lactic acid instead of hydrochloric acid [19]. We found that the expression of IL-4-stimulated macrophages FcγRIIB was upregulated in the acidic microenvironment, and this trend increased with increasing acidity (Fig. 8H–K). In summary, these results indicate that IL-4 enhances the production of lactic acid by enhancing glycolysis in M2 macrophages, promotes the formation of tumor acidic microenvironment, and upregulates the expression of FcγRIIB.

Then we further carried out LDHA knockdown on M2 macrophages. After verifying the knockdown efficiency, the cell line with the lowest LDHA expression was selected (Supplementary Fig. 7A, B), and the expression level of FcγRIIB was significantly decreased after detection (Supplementary Fig. 7C, D). This also suggests that glycolytic-mediated upregulation of FcγRIIB is regulated by endogenous lactic acid.

### Macrophage-derived FcγRIIB inhibits T cell killing effect

**Fig. 9:**
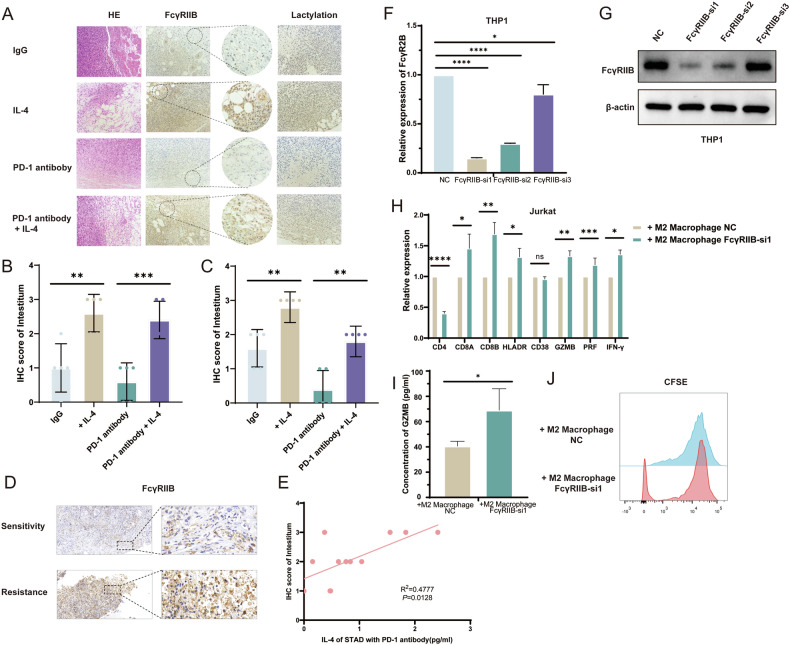
Macrophage-derived FcγRIIB inhibits T cell killing effect. **A** Immunohistochemical staining of xenograft tumors. **B** FcγRIIB immunohistochemical score. **C** Total protein lactated immunohistochemical score. **D** FcγRIIB immunohistochemical staining of clinical samples of immunotherapy patients with gastric cancer. **E** The immunohistochemical score of FcγRIIB was correlated with plasma IL-4 level. **F** The knockdown of FcγRIIB in M2 macrophages was detected by qRT-PCR. **G** The knockdown of FcγRIIB in M2 macrophages was detected by western blot. **H** The immunosuppression of co-cultured Jurkat cells was detected by qRT-PCR. **I** The immunosuppression of co-cultured Jurkat cells was detected by ELISA. **J** Proliferative ability of co-cultured Jurkat cells was detected by CFSE experiments.

Immunohistochemistry was used to detect the expression of FcγRIIB in mouse xenograft tumors (Fig. 9A), and it was found that IL-4 significantly enhanced the expression of FcγRIIB in subcutaneous tumor interstitial tissue (Fig. 9B). Meanwhile, in order to detect the glycolysis of each group in vivo, We also found that IL-4 significantly enhanced total protein lactation in tumor interstitial tissue (Fig. 9C). To further investigate the relationship between the FcγRIIB and immune evasion, we measured the IL-4 expression in serum and FcγRIIB expression of interstitial tissue cells in tumor tissues from the 12 cases of gastric cancer patients (6 from Immunotherapy sensitive group and 6 from Immunotherapy resistant group). It was found that the expression of FcγRIIB was increased in the interstitial tissue of the immunotherapy-resistant group (Fig. 9D). And the results revealed a positive correlation between IL-4 level in serum and FcγRIIB expression of M2 macrophages cells in tumor tissues (Fig. 9E). In order to further confirm the inhibitory effect of FcγRIIB of macrophages in the immune microenvironment, we used siRNA plasmid to knock down FcγRIIB on M2 macrophages derived from THP1 and co-culture them with activated T cells (Fig. 9F, G). qRT-PCR results showed increased expressions of GZMB, IFN-γ, and HLADR in T cells (Fig. 9H), while ELISA results also showed that GZMB expression in T cells was increased (Fig. 9I). CSFE experiment also confirmed that the proliferation ability of T cells was up-regulated after co-culture with FcγRIIB knockdown macrophages (Fig. 9J).

## Discussion

**Fig. 10:**
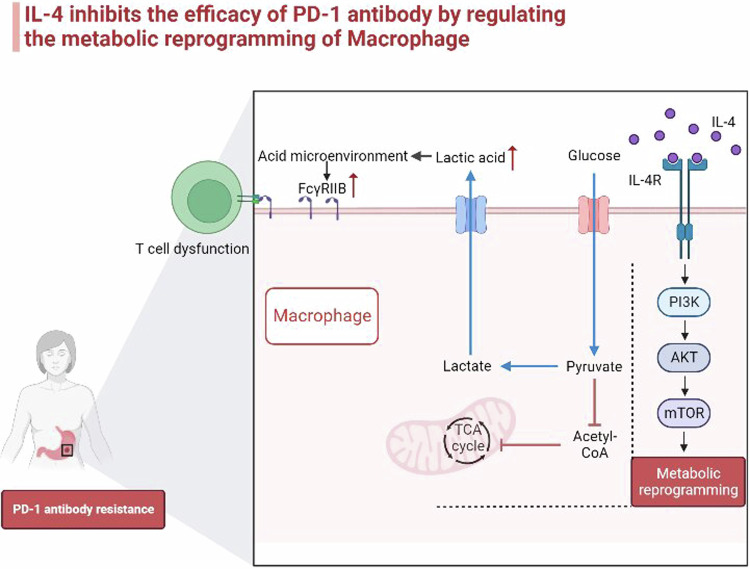
IL-4 inhibits the efficacy of PD-1 antibody by regulating the metabolic reprogramming of macrophage. At the same time of M2 polarization, IL-4 induced metabolic reprogramming of macrophages in gastric cancer microenvironment by up-regulating PI3K/AKT/mTOR signaling pathway. Then the expression of FcγRIIB in M2 macrophages was up-regulated through the acidic microenvironment, ultimately inducing CD8+T cell dysfunction and resistance to PD-1 antibody therapy in gastric cancer.

Our results showed that the expression level of L-4 increased in the plasma of the immunotherapy-resistant group, and patients with high levels of IL-4 had lower objective response rate (ORR) and shorter progression-free survival (PFS), and the effect of IL-4 on inhibiting mouse gastric cancer PD-1 antibody efficacy was verified by e in vivo experiment. In this study, we found that while IL-4 promoted the M2 polarization of macrophages in vitro, it also caused metabolic reprogramming of macrophages in the gastric cancer microenvironment by up-regulating the PI3K/AKT/mTOR signaling pathway: oxidative phosphorylation was inhibited, glycolysis was enhanced, the production of lactic acid increased. In vitro experiments, we found that metabolic reprogramming can promote the production of acidic microenvironment by releasing lactic acid into the tumor microenvironment, and then up-regulate the expression of FcγRIIB on M2 macrophages, leading to CD8^+^T cell dysfunction and PD-1 antibody treatment resistance. The mechanism was verified by xenotransplantation of mouse tumors and clinical samples (Fig. 10).

We believe that in the statistics and treatment of clinical samples, the number of included cases is still insufficient. Meanwhile, the objective response rate (ORR) of gastric cancer patients with immunotherapy combined with chemotherapy included in our study is only 42%, which is still relatively low compared with the published clinical studies on gastric cancer immunotherapy [5]. This may be explained by the bias caused by the late stage and poor disease control in the patients we included who were mostly hospitalized in the ward.

Mismatch repair (MMR), as a factor affecting immunotherapy efficacy, did not show significant differences in the baseline data table, which we hypothesized was due to the small proportion of patients with dMMR: only 4 in each group. In addition, through statistical analysis of metastasis in tumor patients, we found that liver metastasis, lung metastasis, bone metastasis and lymph node metastasis of advanced gastric cancer were not correlated with immunotherapy efficacy, while patients in immunotherapy-resistant group had more abdominal and pelvic metastasis. We speculated that peritoneal metastasis of gastric cancer was often complicated by peritoneal metastasis. The presence of the peritoneo-plasma barrier results in poor efficacy of macromolecular targeted drugs.

In fact, there are more indicators that should be counted, such as MSI and TMB, which are generally associated with immunotherapy efficacy. However, only a small number of patients in our statistical cases got genetic testing, so these indicators were not included, and only the correlation between IL-4, IL-4R and MSI, TMB was analyzed in public data.

In our study, in the analysis of cytokines in immunotherapy-resistant patients, we found that plasma levels of cytokines IL-1β, IL-2, IL-4, IL-5 and IL-22 increased in immunotherapy-resistant patients, and only IL-4 expression level increased after secondary drug resistance in patients during follow-up. Therefore, we chose IL-4 for follow-up study. In fact, IL-1β is also a well-recognized cytokine that has an inhibitory effect on the immune microenvironment. It has been confirmed in breast cancer that IL-1β can promote tumor progression by recruiting tumor-associated macrophages, while blocking IL-1β can inhibit tumor immune escape [20]. This suggests that IL-1β may have a synergistic effect with IL-4 in promoting immunotherapy resistance of gastric cancer through macrophages. However, IL-5 and IL-22 have not been associated with tumor immunotherapy, and their effects still need to be further studied. For IL-2, although it is a cytokine that activates the immune environment and has been proven to promote the proliferation and killing of T cells in anti-tumor immunity [21], and has been approved for anti-tumor therapy at present. In our clinical data, it has increased in the immunotherapy-resistant group, and we speculate that it may caused by the production of cytokine amoung highly proliferating tumor cells in immunotherapy-resistant patients.

In order to confirm that it is high levels of IL-4 that inhibit the efficacy of immunotherapy, rather than high proliferation of tumor cells after drug resistance and excessive production of IL-4, we verified in animal models that IL-4 can inhibit the efficacy of mouse PD-1 antibody. Since in vitro experiments cannot completely simulate the anti-tumor effect of PD-1 antibody in related studies of tumor immunotherapy, even organoid models are difficult to become effective evidence for evaluating the efficacy of immunotherapy [22], so animal experiments are particularly important. We consider that immunotherapy is different from other anticancer drugs in its mechanism of action. Owing to the lack of T cells in the immune system of nude mice, the killing effect of PD-1 antibody after activating the immune system cannot be simulated. In the model construction of animal experiments, we chose mice that were close to the human immune system as much as possible [23], instead of the human tumor xenotransplantation model (PDX) commonly used in animal experiments. Therefore, we used MFC cells to construct xenograft tumors in the construction of animal models [24], and 615 mice were used as tumor-bearing mice to improve the success rate of implantation of transplanted tumors [25], although MFC cells were confirmed as squamous cell cells in sequencing. In fact, due to the presence of the anterior stomach without a glandular structure in the mouse stomach structure, spontaneous gastric cancer induced by carcinogens and various mouse gastric cancer cell lines are often proved to be squamous carcinoma, rather than the clinically common adenocarcinoma.

In vitro functional experiments, our exploration of metabolic reprogramming during M2 polarization in macrophages focused on glucose metabolism, mainly based on our non-targeted metabolic sequencing results, which we verified in vitro experiments. However, these partial results are different from the current view of macrophage metabolism: M1 macrophages are mainly based on glycolysis metabolic pathway while M2 macrophages are mainly based on fatty acid oxidative metabolic pathway [26]. First of all, the overall metabolic trend of macrophages during activation shifts from mitochondrial oxidative metabolism to glycolysis. Studies have shown that the enhancement of glycolysis was also observed after IL-4 stimulated mouse bone marrow-derived macrophages [13]. In addition, it was found that THP1 cell line has a high glycolysis intensity and has its unique metabolic characteristics [27]. In fact, in the results of non-targeted metabolic sequencing, it was also found that some products of fatty acid metabolism were increased in M2 macrophages, suggesting an active fatty acid metabolic pathway.

We selected the PI3K/AKT/mTOR signaling pathway that regulates metabolic reprogramming of macrophages, mainly because we found upregulation of the mTOR signaling pathway in RNA-seq of THP1 after IL-4 stimulation, and mTOR signaling is usually activated in tumors. It has been widely studied in the metabolic reprogramming of tumor cells and immune cells [13, 28]. mTOR signaling pathway can normally activate GLUT1 [29], a key glycolysis protein, which can enhance the Warburg effect of tumor cells through the transcription factors HIF1α and MYC. mTOR signaling pathway has also been proven to upregulate the expression of HK2, a key glycolysis enzyme [30]. At the same time, by using mTORC1/C2 inhibitors, we demonstrated that increased glycolysis and lactic acid production during M2 macrophage are mediated by the PI3K/AKT/mTOR signaling pathway.

In our study, qRT-PCR and western blot showed that the expression of multiple immune checkpoint ligands on the surface of macrophages stimulated by IL-4 was upregulated, such as PD-L1, PD-L2, GAL9, HMGB1, FGL1. Although the mechanism and its correlation with immunotherapy resistance of gastric cancer were not thoroughly studied in the end. Based on previous reports on immune checkpoint ligands, it can still be used as evidence for the negative regulatory role of M2 macrophages in the tumor immune microenvironment. At present, a number of studies have reported that CD14^+^ or CD68^+^ macrophages have been observed to up-regulate the expression of PD-L1 in a variety of cancer tissues, including hepatocellular carcinoma, melanoma and ovarian cancer [31, 32].

Through the analysis of multiple sequencing data sets, our study found a molecule on macrophages that is related to the efficacy of immunotherapy. As a molecule that mediates immunosuppressive signals mainly expressed on B cells and macrophages, the effect of inhibiting signal transduction by FcγRIIB on B cells has been extensively studied [33]. However, the mechanism of how FcγRIIB on macrophages regulates T cell killing and immunotherapy efficacy during anti-tumor immunity is still poorly understood. It has been observed that high expression of FcγRIIB can cause dysfunction of CD8^+^T cells and weaken their response to immune checkpoint inhibitors [34]. And it has been reported that FcγRIIB can be expressed on the surface of T cells and play an immunosuppressive role as an immune checkpoint [35], and it has also been demonstrated that FcγRIIB on macrophages can be captured by T cells through intercellular transfer, and then inhibit the proliferation and cytotoxic effects of T cells [36]. We hypothesize that IL-4 plays a role through a similar mechanism after upregulating the expression of FcγRIIB on macrophages. However, our study on the mechanism of the influence of FcγRIIB expression still needs to be further explored.

In our study, FcγRIIB knockdown macrophage cell lines were constructed in vitro and co-cultured with T cells to find that the killing effect of T cells was restored compared with the control group. However, there are still shortcomings in our research on FcγRIIB. In the future, we will consider knocking out macrophage FcγRIIB in vivo experiments to observe the efficacy of immunotherapy, so as to prove the molecule FcγRIIB is an important factor affecting the efficacy of immunotherapy.

In summary, this study takes the lead in finding that IL-4 is associated with immunotherapy resistance in gastric cancer, and to reveal the molecular mechanism of the metabolic reprogramming of macrophages mediated by IL-4. This study provides a new and effective predictor for the immunotherapy of gastric cancer, and provides a potential therapeutic target and theoretical basis for improving the clinical efficacy of PD-1 antibody therapy.

## Method

### Gastric cancer immunotherapy samples

This study counted gastric cancer patients who received palliative first-line treatment using immunotherapy combined with chemotherapy in our center from January 2020 to June 2022. All patients had distant metastases. A total of 106 patients were enrolled, whose efficacy was evaluated using iRECIST (immunotherapeutic Efficacy Evaluation Criteria for solid tumors) based on enhanced CT results once every 6–8 weeks. If patients had a history of PR, they were included in the sensitive group; otherwise, they were included in the drug-resistant group. The longest follow-up time was 1 year. We collected the basic information, tumor metastasis, laboratory test results, tumor pathology and immunohistochemical results of these patients.

### Cell line

Mouse gastric cancer cell lines MFC, human monocyte leukemia cell lines THP1 and U937, and human T-cell leukemia cell lines Jurkat were all purchased from the cell bank of Chinese Academy of Sciences, and cultured using 1640 culture containing 10%FBS and 1× cyan-streptomycin.

### Animal experiment model

In animal experiments, four-week male 615 mice were subcutaneously injected with 1 × 10^6^ MFC mouse gastric cancer cells in the posterior part of the right axilla. The weight and size of the tumor were recorded four days after the subcutum injection, once every three days. PD-1 antibody was injected 7 days after subcutaneous tumor injection, 125 μg biw tumor intraperitoneal injection. IL-4 was injected twice a week, the day before immunotherapy, 1 μg biw tumor local subcutaneous injection. On the 16th day after subcutaneous tumor transplantation, the mice were killed after blood collection.

### Public database and analysis tools

1. The Cancer Genome Atlas (TCGA) : The cancer genome atlas includes clinical data of various human tumors, genomic variation, mRNA expression, miRNA expression, methylation and other data.
2. GSE163558: 10 tissue samples from 6 patients with gastric cancer with different clinical stages were included in the study. Including 3 primary gastric cancer samples, 1 paracancer normal tissue, 2 lymph node metastases, 1 ovarian metastases, 1 peritoneal metastases, and 2 liver metastases of gastric cancer. Tumor specimens were collected through surgery and processed for single-cell sequencing. Finally, 10 cases of RNA-seq data were uploaded to GEO database for analysis [37].

### Macrophage polarization

THP1/U937 cells at logarithmic growth stage were centrifuged, and serum-free medium 1640 was used to resuscitate the cells. 185 ng/ml PMA (dissolved with DMSO) was added for 6 h to induce differentiation towards macrophages. Then, under the presence of PMA, IFN-γ(20 ng/ml) and LPS(100 ng/ml) were added for 48 h to stimulate it to polarize towards M1 macrophage. IL-4(20 ng/ml) and IL-13(20 ng/ml) were added for 48 hours to stimulate it to polarize towards M2 macrophage, and follow-up detection was performed.

### Macrophages co-cultured with T cells

1 × 10^6^ macrophages were inoculated with PMA and with/without IL-4 stimulation. Under the presence of drugs, 1 × 10^6^ activated Jurkat cells/PBMC derived T cells from gastric cancer patients were added (Jurkat cells or PBMC were added with 25 μl/ml CD3/28 T cell Activator and 20 ng/ml IL-2 stimulation [38, 39] for 72 h). After co-culture for 48 h, T cells were collected using T cell Isolation kit. After centrifugation, follow-up detection was conducted.

### Survival analysis

Progression-free Survival (PFS) was defined as the time from initiation of immunotherapy to imaging assessment of disease Progression. Kaplan-Meier survival analysis, Log-rank test and COX regression analysis were performed. Patients were grouped according to age, gender, treatment plan, MMR, primary site, Lauren type and other factors, and COX regression model was used for subgroup analysis.

### Statistical methods

Continuity variables were expressed as mean ± standard deviation. T test was used to compare the data of two groups of continuous variables. The data of multiple groups of continuous variables were compared by one-way analysis of variance. The data that did not meet the parameter test were analyzed by Kruskal–Wallis method. Categorical variables were analyzed by Fisher method. The survival curve method (Kaplan-Meier) was used to compare PFS among different groups. Data analysis and charting were performed using GraphPad Prism (Version 9.0.0) and SPSS (Version 26.0.0). *P* < 0.05 indicates a statistically significant difference.

### Supplementary information

Supplementary data Figure legend

Supplementary Figure 1-7

Western blot

## Data Availability

The data analyzed in this study were obtained from The Cancer Genome Atlas and Gene Expression Omnibus (GEO) at GSE163558. The data of gastric cancer patients with immunotherapy generated in this study are not publicly available due to patient privacy requirements but are available upon reasonable request from the corresponding author.

## Code availability

The code used in the study is available upon reasonable request from the corresponding author.
