# Supplementary material for: IL-4/IL-4R axis signaling drives resistance to immunotherapy by inducing the upregulation of Fcγ receptor IIB in M2 macrophages
Source: Cell Death Dis. 2024 Jul 13;15(7):500. doi: 10.1038/s41419-024-06875-4 (PMC11246528; doi:10.1038/s41419-024-06875-4)
Supplement: Supplementary file 1 — Supplementary data Figure legend [file 41419_2024_6875_MOESM1_ESM.docx]

**Supplementary data**

**Supplementary Fig. 1 IL-4/IL-4R axis affect the prognosis of patients with gastric cancer**

A Analysis of IL-4R and OS in patients with gastric cancer by TCGA data

B Analysis of IL-4R and PFS in patients with gastric cancer by TCGA data

C The expression of IL-4 in gastric cancer and adjacent normal tissues was analyzed by TCGA data

D The expression of IL-4R in gastric cancer and adjacent normal tissues was analyzed by TCGA data

E Analysis of IL-4 expression and MSIscore in gastric cancer by TCGA data

F Analysis of IL-4 expression and TMB in gastric cancer by TCGA data

G Analysis of IL-4R expression and MSIscore in gastric cancer by TCGA data

H Analysis of IL-4R expression and TMB in gastric cancer by TCGA data

**Supplementary Fig. 2 Immune cell infiltration of gastric cancer and normal tissue**

A The thermogram of immune cell infiltration in gastric cancer and adjacent normal tissue analyzed by TCGA data

B The box diagram of immune cell infiltration in gastric cancer and adjacent normal tissue analyzed by TCGA data

C TCGA data analysis of immune cell infiltration and prognosis of gastric cancer patients

**Supplementary Fig. 3 IL-4/IL-4R axis and macrophage infiltration**

A IL-4R and macrophage infiltration in TCGA data were analyzed by xCELL

B IL-4R and macrophage infiltration in TCGA data were analyzed by TIMER

**Supplementary Fig. 4 Activation of the downstream pathway of IL-4/IL-4R axis in GSE163558**

A The expression of PI3K in each cell subpopulation in single cell sequencing

B The expression of AKT1 in each cell subpopulation in single cell sequencing

C The expression of AKT2 in each cell subpopulation in single cell sequencing

D The expression of STAT6 in each cell subpopulation in single cell sequencing

E The expression of PI3K in each macrophage subpopulation in single cell sequencing

F The expression of AKT1 in each macrophage subpopulation in single cell sequencing

G The expression of AKT2 in each macrophage subpopulation in single cell sequencing

H The expression of STAT6 in each macrophage subpopulation in single cell sequencing

**Supplementary Fig. 5 IL-4 stimulates the polarization of monocyte-derived macrophages towards M2**

A PMA, IL-4, IL-13, LPS, IFN-γ were used to stimulate human leukemia mononuclear cell lines THP1 and U937

B Polarization condition in THP1 after IL-4 stimulation was detected by qRT-PCR

C Polarization condition in U937 after IL-4 stimulation was detected by qRT-PCR

D Polarization condition in THP1 after IL-4, IL-13 stimulation was detected by qRT-PCR

E Polarization condition in THP1 after IL-4 stimulation was detected by flow cytometry

F Polarization condition in U937 after IL-4 stimulation was detected by flow cytometry

G The expressions of F4/80 and CD206 in mouse subcutaneous tumor were detected by immunofluorescence

H Plasma IL-4 level of mouse was detected by ELISA

**Supplementary Fig. 6 Effect of type II IL-4R on activation of PI3K-AKT-mTOR pathway**

A The knockdown of IL-13Rα1 in M2 macrophages was detected by qRT-PCR

B Western blot analysis of PI3K/AKT/mTOR signaling pathway in THP1 with IL-4 stimulation after IL-13Rα1 knockdown

**Supplementary Fig. 7 Regulation of macrophage FcγRIIB by glycolysis is dependent on endogenous lactic acid**

A The knockdown of LDHA in M2 macrophages was detected by qRT-PCR

B The knockdown of LDHA in M2 macrophages was detected by Western blot

C The expression of FcγRIIB in M2 macrophages was detected by qRT-PCR

D The expression of FcγRIIB in M2 macrophages was detected by Western blot
