# Supplementary material for: IL-4/IL-4R axis signaling drives resistance to immunotherapy by inducing the upregulation of Fcγ receptor IIB in M2 macrophages
Source: Cell Death Dis. 2024 Jul 13;15(7):500. doi: 10.1038/s41419-024-06875-4 (PMC11246528; doi:10.1038/s41419-024-06875-4)
Supplement: Supplementary file 2 — Supplementary Figure 1-7 [file 41419_2024_6875_MOESM2_ESM.pdf]

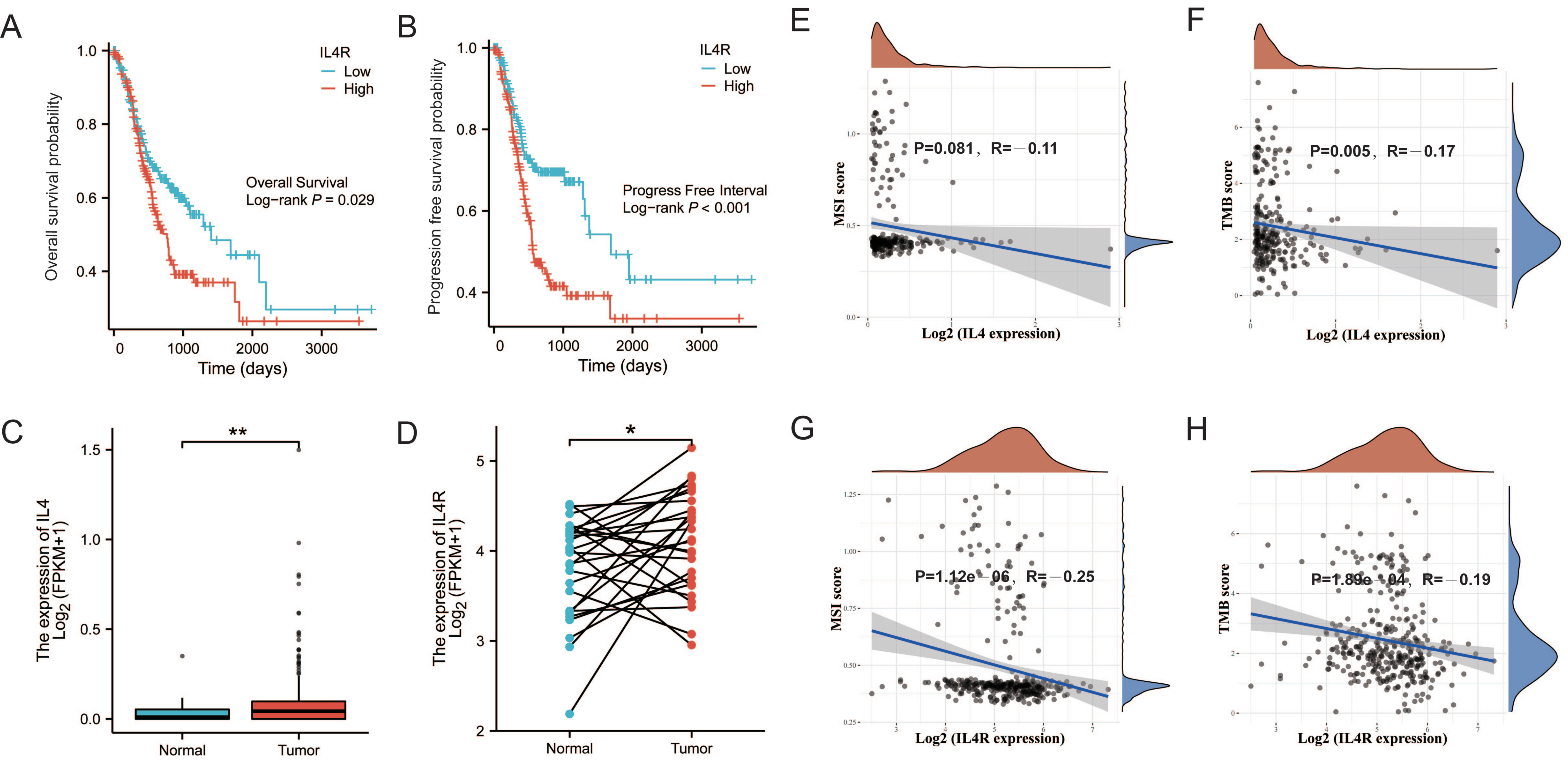
A
IL4R
Low
High
Overall Survival
Log−rank P = 0.029
Overall survival probability
Time (days)
B
IL4R
Low
High
Progress Free Interval
Log−rank P < 0.001
Progression free survival probability
Time (days)
C
**
The expression of IL4
Log2 (FPKM+1)
Normal
Tumor
D
*
The expression of IL4R
Log2 (FPKM+1)
Normal
Tumor
E
P=0.081, R=−0.11
MSI score
Log2 (IL4 expression)
F
P=0.005, R=−0.17
TMB score
Log2 (IL4 expression)
G
P=1.12e−06, R=−0.25
MSI score
Log2 (IL4R expression)
H
P=1.89e−04, R=−0.19
TMB score
Log2 (IL4R expression)

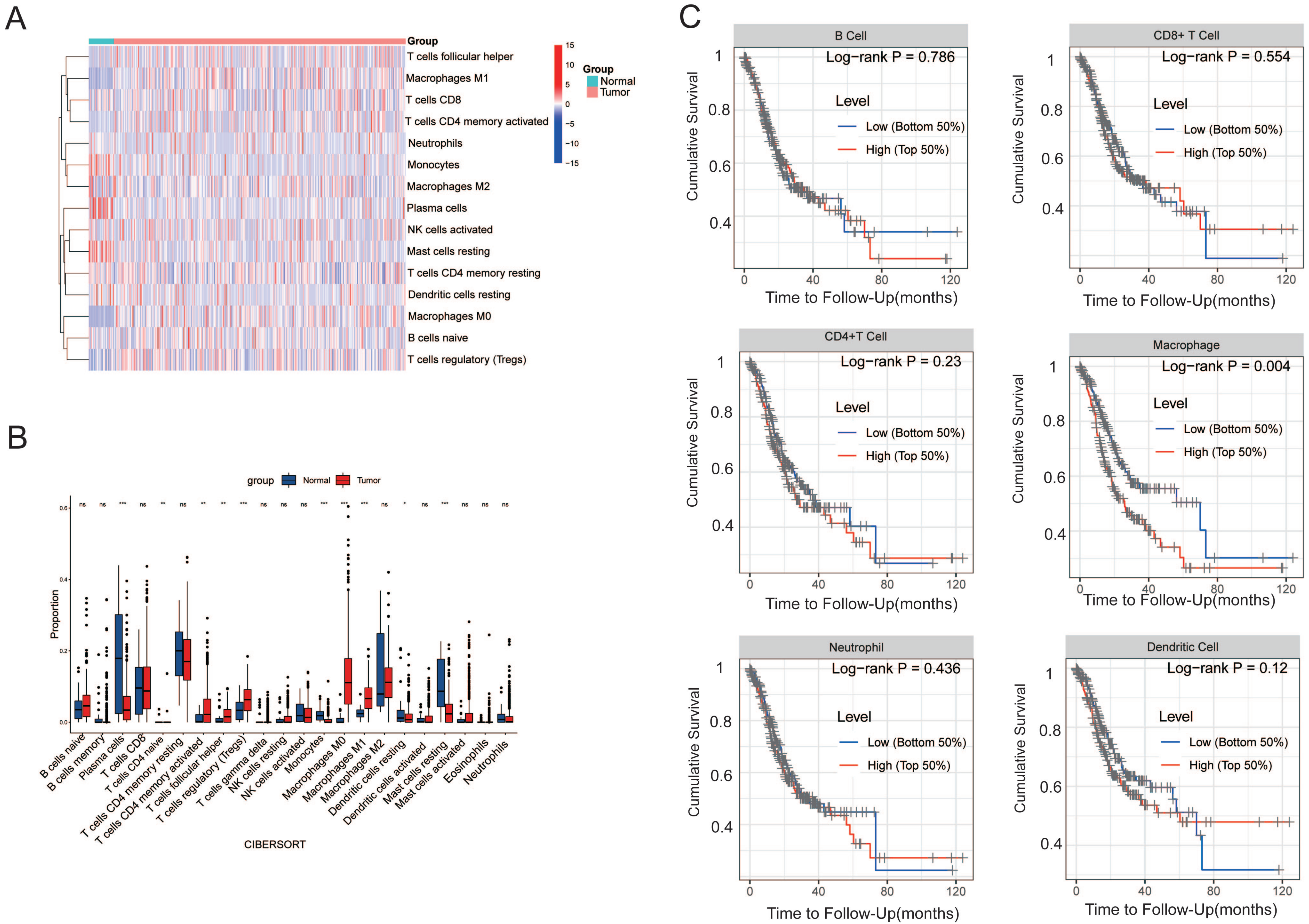
A
Group
T cells follicular helper
Macrophages M1
T cells CD8
T cells CD4 memory activated
Neutrophils
Monocytes
Macrophages M2
Plasma cells
NK cells activated
Mast cells resting
T cells CD4 memory resting
Dendritic cells resting
Macrophages M0
B cells naive
T cells regulatory (Tregs)
Group
Normal
Tumor
B
group
Normal
Tumor
Proportion
CIBERSORT
B cells naive
B cells memory
Plasma cells
T cells CD8
T cells CD4 naive
T cells CD4 memory resting
T cells CD4 memory activated
T cells follicular helper
T cells regulatory (Tregs)
T cells gamma delta
NK cells resting
NK cells activated
Monocytes
Macrophages M0
Macrophages M1
Macrophages M2
Dendritic cells resting
Dendritic cells activated
Mast cells resting
Mast cells activated
Eosinophils
Neutrophils
C
B Cell
Log−rank P = 0.786
CD8+ T Cell
Log−rank P = 0.554
CD4+T Cell
Log−rank P = 0.23
Macrophage
Log−rank P = 0.004
Neutrophil
Log−rank P = 0.436
Dendritic Cell
Log−rank P = 0.12
Level
Low (Bottom 50%)
High (Top 50%)
Cumulative Survival
Time to Follow-Up(months)

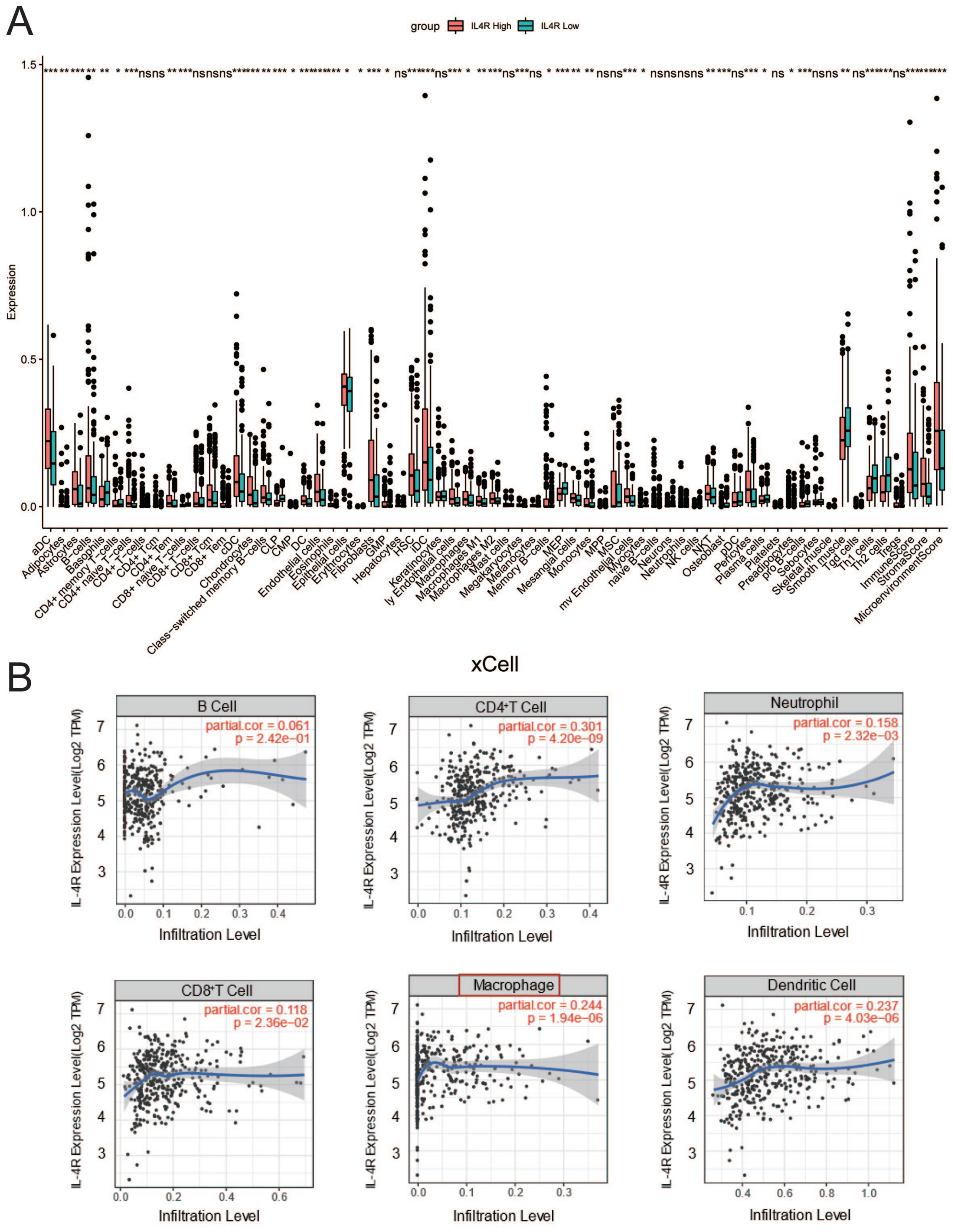

A
group IL4R High IL4R Low
Expression
1.5
1.0
0.5
0.0
aDC
Adipocytes
Astrocytes
B-cells
Basophils
CD4+ memory T-cells
CD4+ naive T-cells
CD4+ T-cells
CD4+ Tcm
CD4+ Tem
CD8+ naive T-cells
CD8+ T-cells
CD8+ Tcm
CD8+ Tem
cDC
Chondrocytes
Class-switched memory B-cells
CLP
CMP
DC
Endothelial cells
Eosinophils
Epithelial cells
Erythrocytes
Fibroblasts
GMP
Hepatocytes
HSC
iDC
Keratinocytes
ly Endothelial cells
Macrophages
Macrophages M1
Macrophages M2
Mast cells
Megakaryocytes
Melanocytes
Memory B-cells
MEP
Mesangial cells
Monocytes
MPP
MSC
mv Endothelial cells
Myocytes
naive B-cells
Neurons
Neutrophils
NK cells
NKT
Osteoblast
pDC
Pericytes
Plasma cells
Platelets
Preadipocytes
pro B-cells
Sebocytes
Skeletal muscle
Smooth muscle
Tgd cells
Th1 cells
Th2 cells
Tregs
ImmuneScore
StromaScore
MicroenvironmentScore
B
xCell
B Cell
partial.cor = 0.061
p = 2.42e-01
CD4+T Cell
partial.cor = 0.301
p = 4.20e-09
Neutrophil
partial.cor = 0.158
p = 2.32e-03
CD8+T Cell
partial.cor = 0.118
p = 2.36e-02
Macrophage
partial.cor = 0.244
p = 1.94e-06
Dendritic Cell
partial.cor = 0.237
p = 4.03e-06
IL-4R Expression Level(Log2 TPM)
Infiltration Level

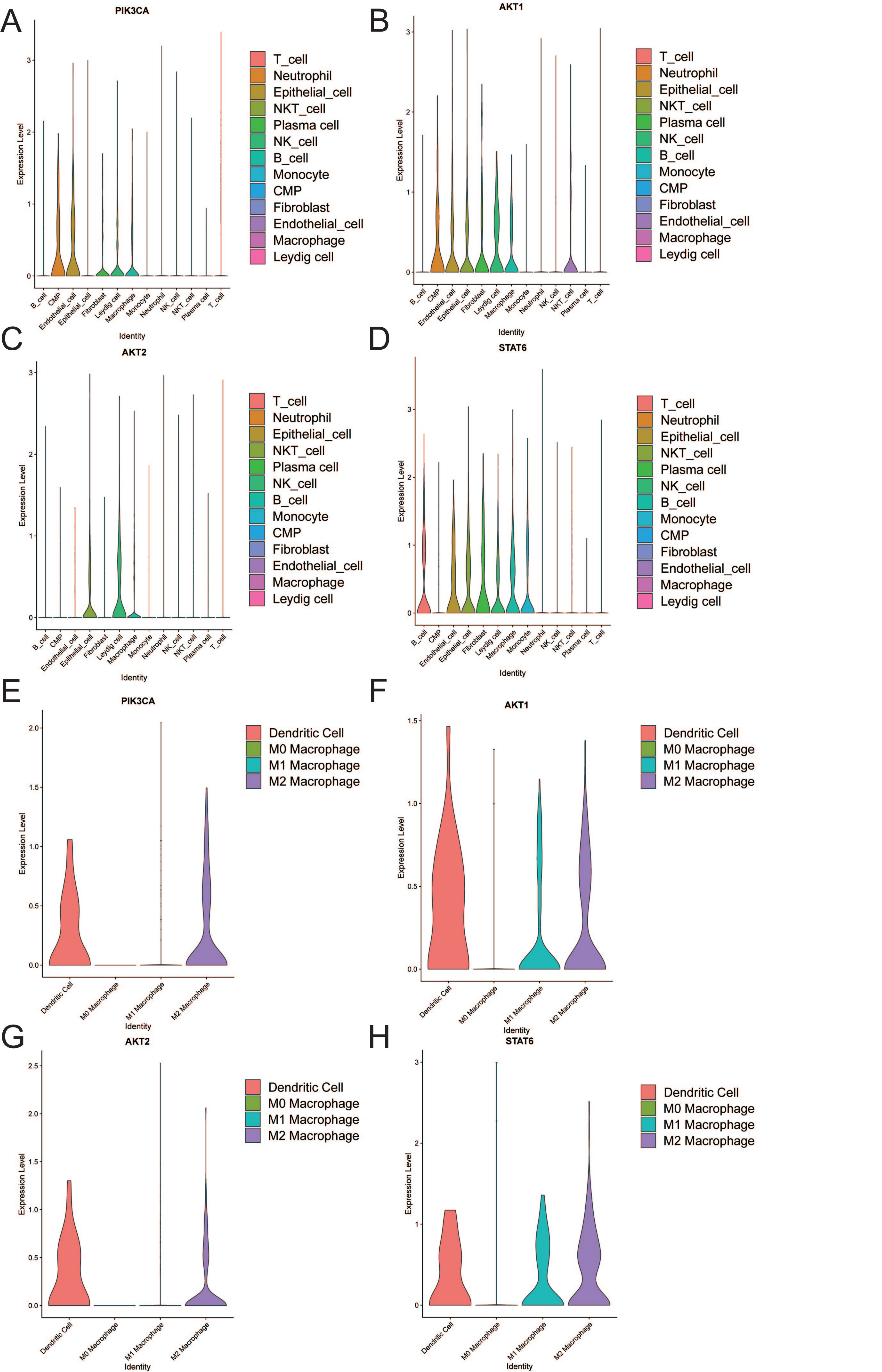
A
PIK3CA
Expression Level
Identity
B_cell
CMP
Endothelial_cell
Epithelial_cell
Fibroblast
Leydig cell
Macrophage
Monocyte
Neutrophil
NK_cell
NKT_cell
Plasma cell
T_cell
T_cell
Neutrophil
Epithelial_cell
NKT_cell
Plasma cell
NK_cell
B_cell
Monocyte
CMP
Fibroblast
Endothelial_cell
Macrophage
Leydig cell
B
AKT1
C
AKT2
D
STAT6
E
PIK3CA
Dendritic Cell
M0 Macrophage
M1 Macrophage
M2 Macrophage
F
AKT1
G
AKT2
H
STAT6

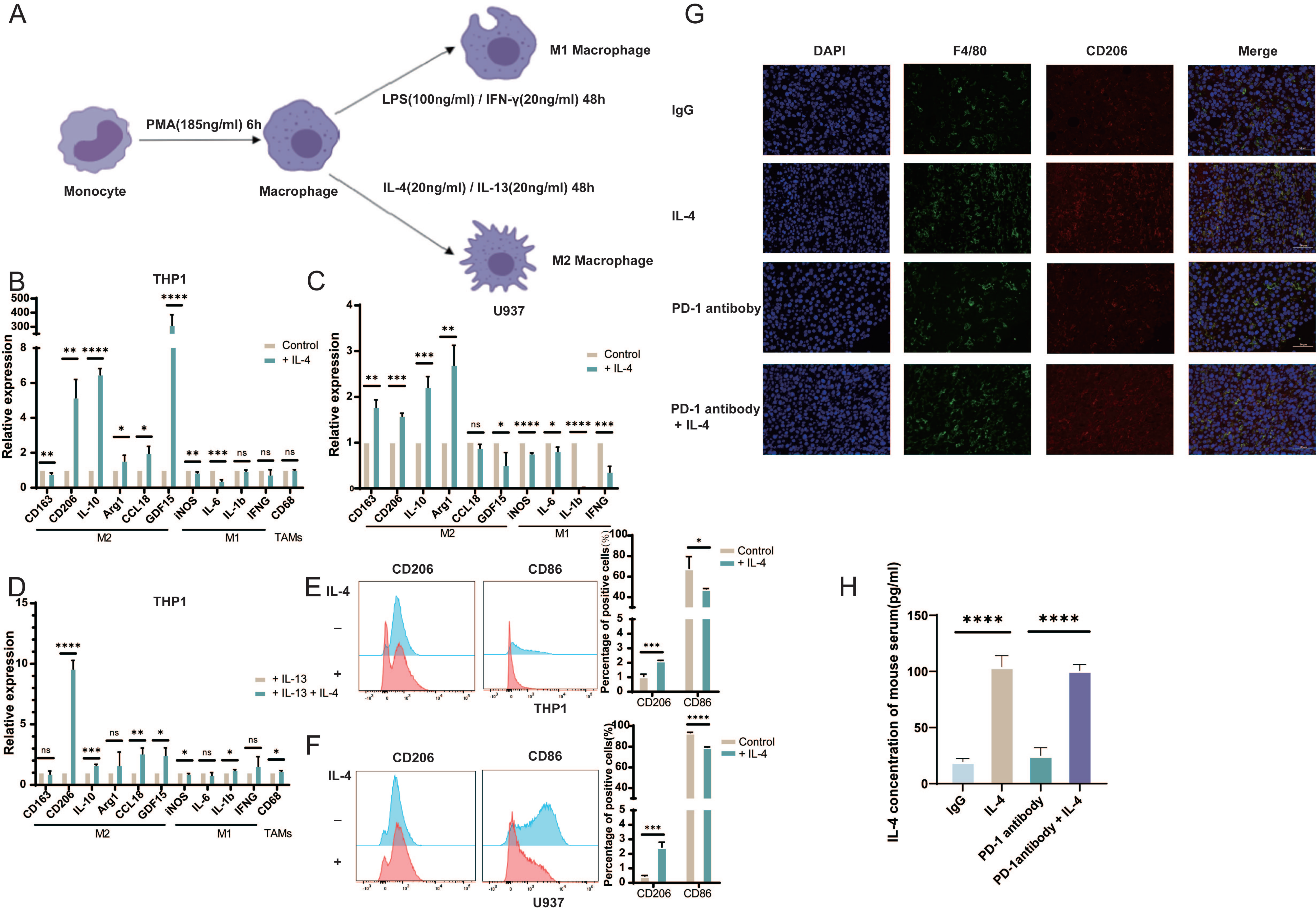
A
Monocyte
PMA(185ng/ml) 6h
Macrophage
LPS(100ng/ml) / IFN-γ(20ng/ml) 48h
M1 Macrophage
IL-4(20ng/ml) / IL-13(20ng/ml) 48h
M2 Macrophage
B
THP1
Relative expression
Control
+ IL-4
CD163 CD206 IL-10 Arg1 CCL18 GDF15 iNOS IL-6 IL-1b IFNG CD68
M2
M1
TAMs
C
U937
Relative expression
Control
+ IL-4
CD163 CD206 IL-10 Arg1 CCL18 GDF15 iNOS IL-6 IL-1b IFNG
M2
M1
D
THP1
Relative expression
+ IL-13
+ IL-13 + IL-4
CD163 CD206 IL-10 Arg1 CCL18 GDF15 iNOS IL-6 IL-1b IFNG CD68
M2
M1
TAMs
E
IL-4
CD206
CD86
THP1
Percentage of positive cells(%)
Control
+ IL-4
CD206 CD86
F
IL-4
CD206
CD86
U937
Percentage of positive cells(%)
Control
+ IL-4
CD206 CD86
G
DAPI
F4/80
CD206
Merge
IgG
IL-4
PD-1 antiboby
PD-1 antibody + IL-4
H
IL-4 concentration of mouse serum(pg/ml)
IgG
IL-4
PD-1 antibody
PD-1antibody + IL-4

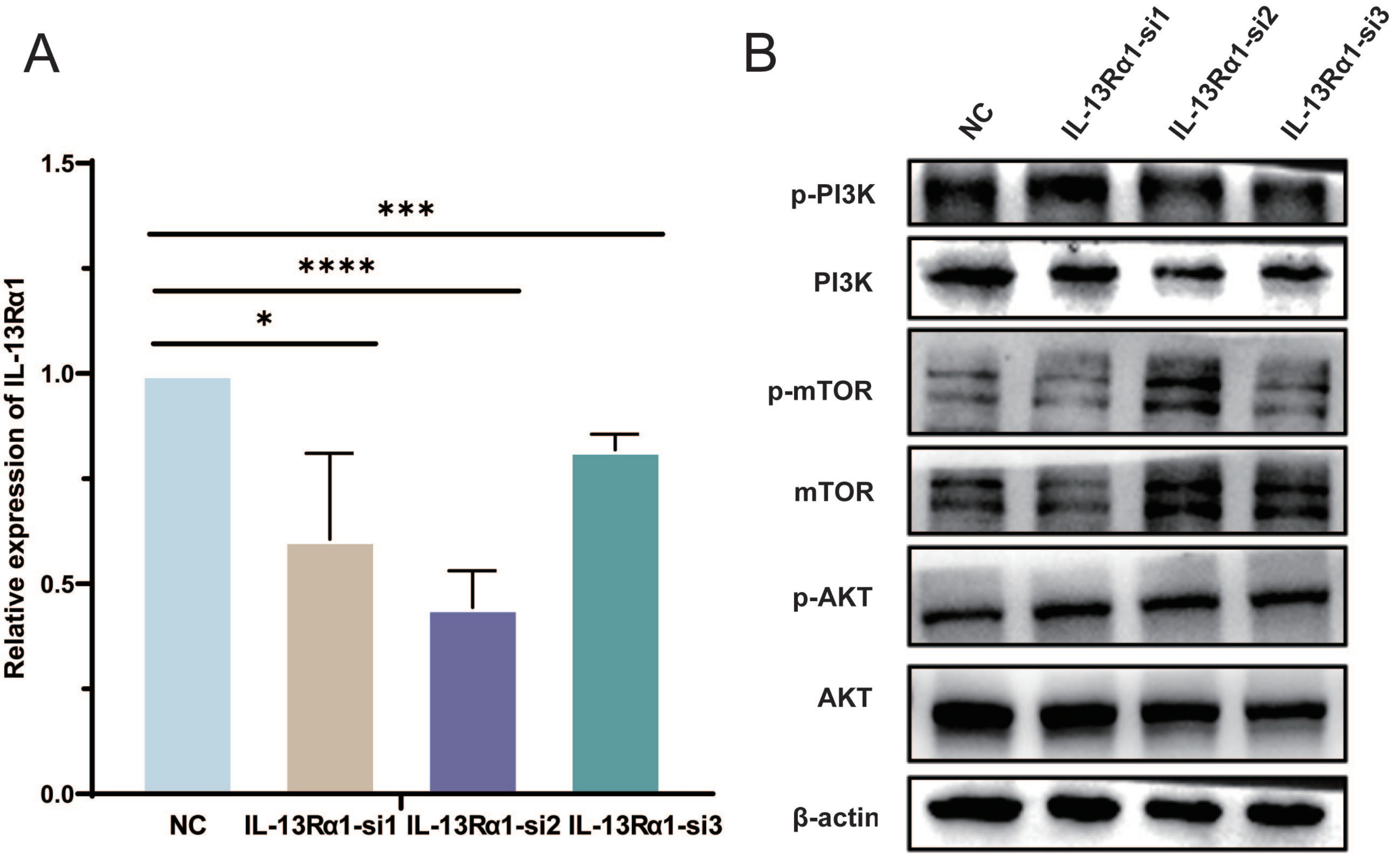
A
B
Relative expression of IL-13Rα1
1.5
1.0
0.5
0.0
***
****
*
NC
IL-13Rα1-si1
IL-13Rα1-si2
IL-13Rα1-si3
p-PI3K
PI3K
p-mTOR
mTOR
p-AKT
AKT
β-actin

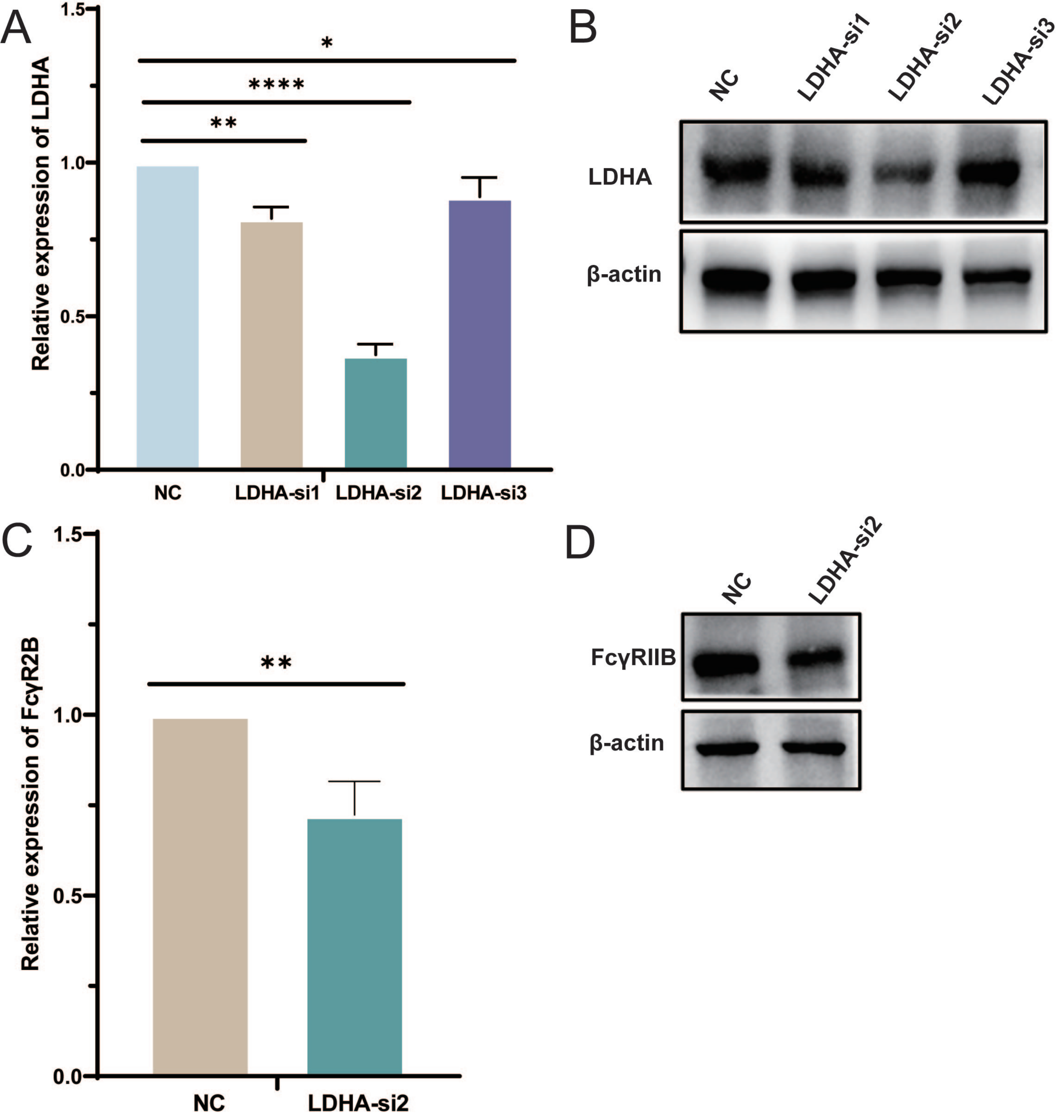
A
Relative expression of LDHA
1.5
1.0
0.5
0.0
*
****
**
NC
LDHA-si1
LDHA-si2
LDHA-si3
B
NC
LDHA-si1
LDHA-si2
LDHA-si3
LDHA
β-actin
C
Relative expression of FcγR2B
1.5
1.0
0.5
0.0
**
NC
LDHA-si2
D
NC
LDHA-si2
FcγRIIB
β-actin
