# Supplementary material for: IL-4/IL-4R axis signaling drives resistance to immunotherapy by inducing the upregulation of Fcγ receptor IIB in M2 macrophages
Source: Cell Death Dis. 2024 Jul 13;15(7):500. doi: 10.1038/s41419-024-06875-4 (PMC11246528; doi:10.1038/s41419-024-06875-4)

Fig 5B

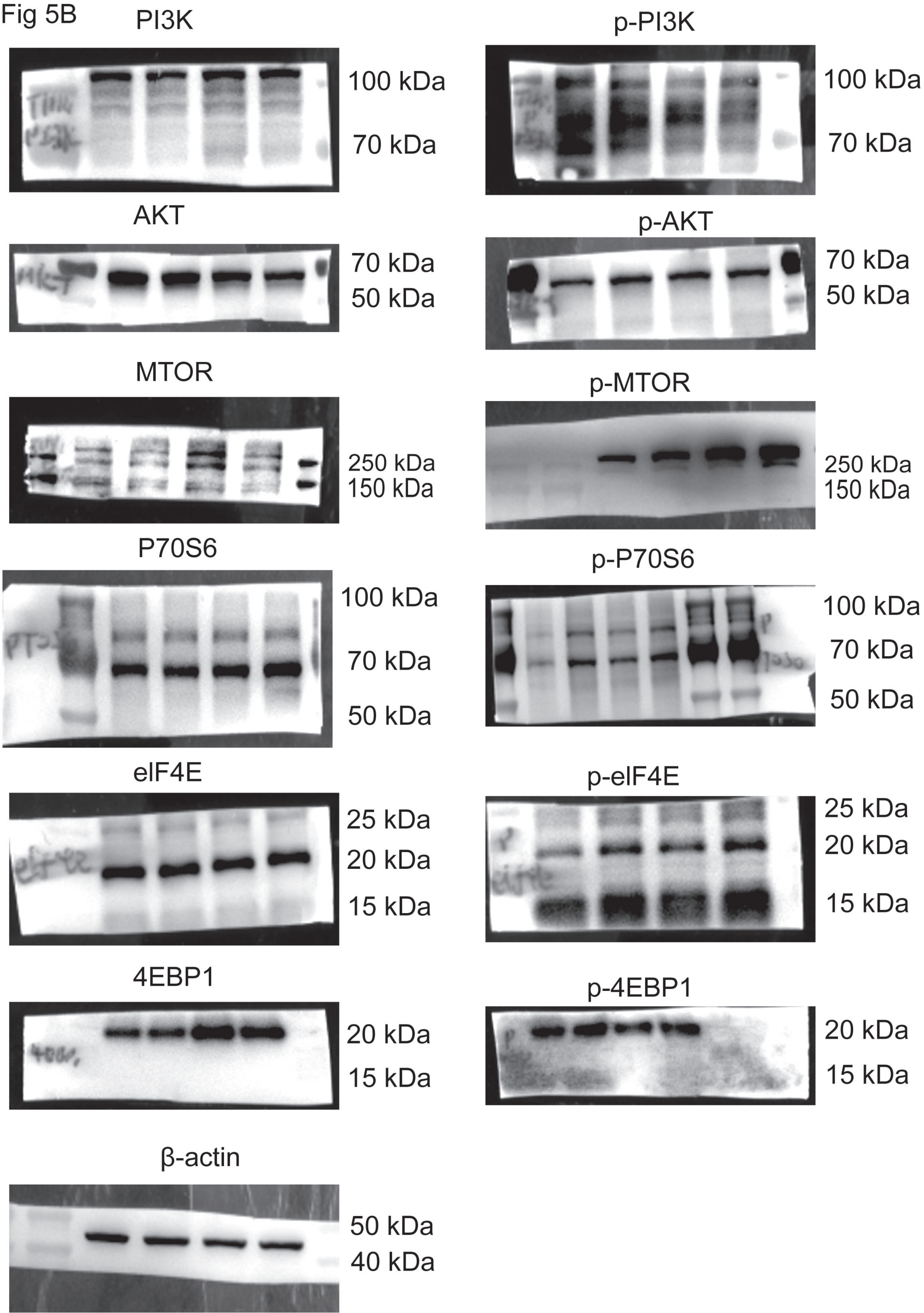
PI3K
100 kDa
70 kDa
p-PI3K
100 kDa
70 kDa
AKT
70 kDa
50 kDa
p-AKT
70 kDa
50 kDa
MTOR
250 kDa
150 kDa
p-MTOR
250 kDa
150 kDa
P70S6
100 kDa
70 kDa
50 kDa
p-P70S6
100 kDa
70 kDa
50 kDa
eIF4E
25 kDa
20 kDa
15 kDa
p-eIF4E
25 kDa
20 kDa
15 kDa
4EBP1
20 kDa
15 kDa
p-4EBP1
20 kDa
15 kDa
β-actin
50 kDa
40 kDa

Fig 6C

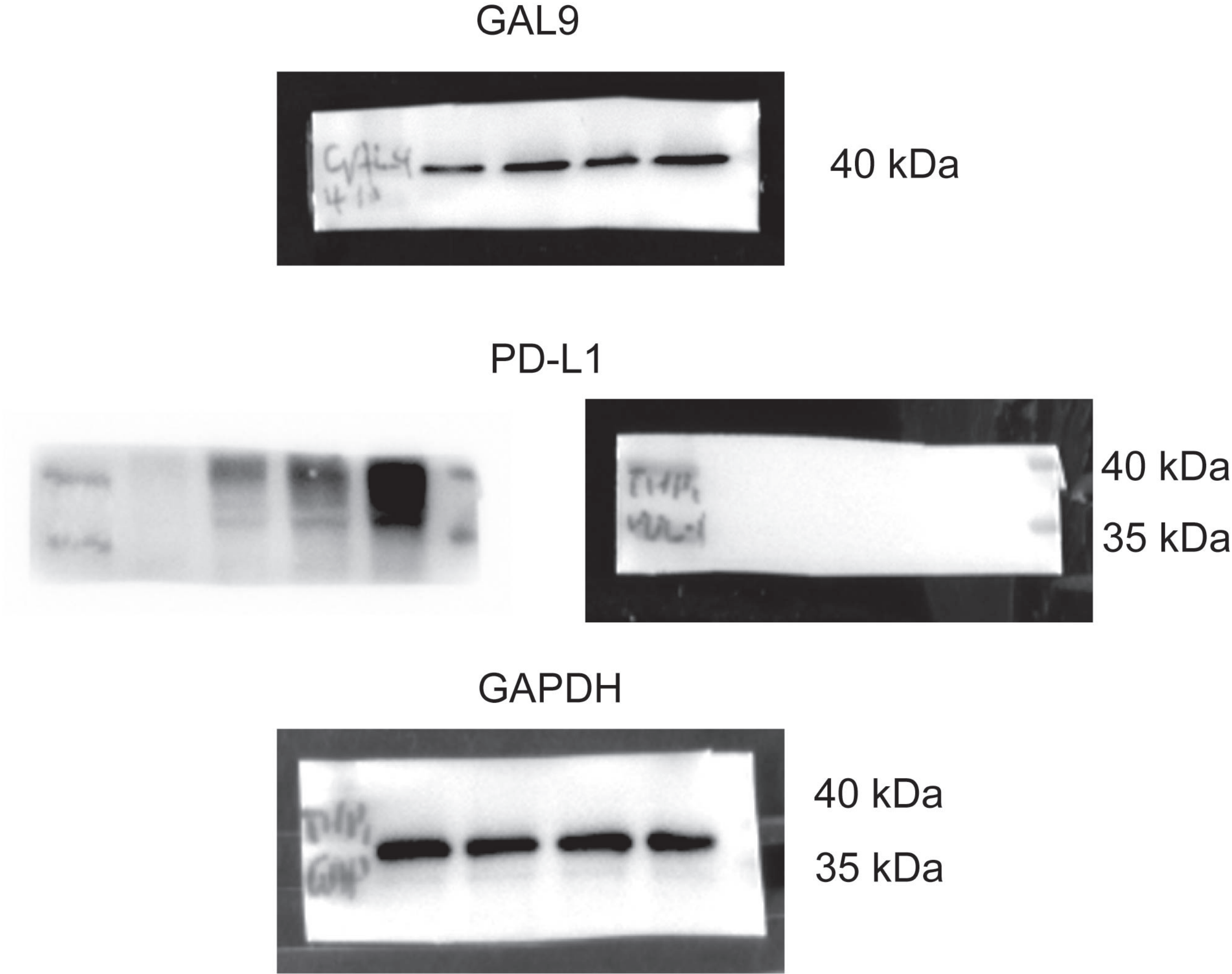

Fig 7B

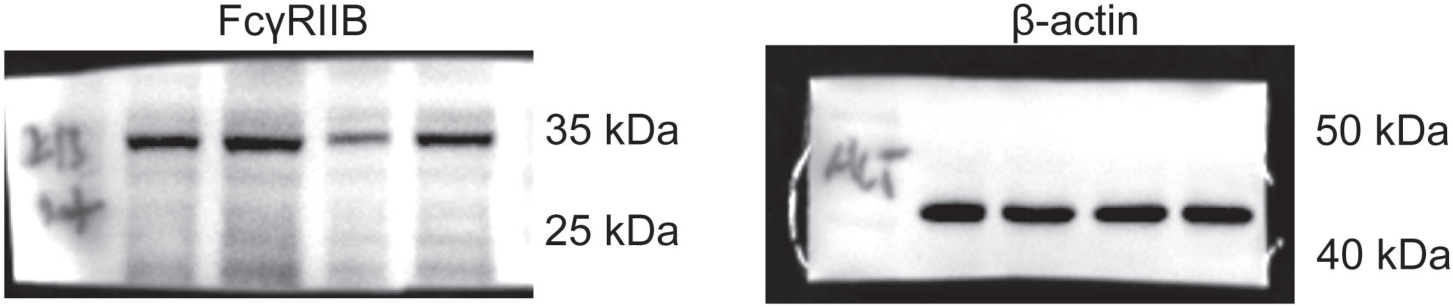
FcγRIIB
35 kDa
25 kDa
β-actin
50 kDa
40 kDa

Fig 7F

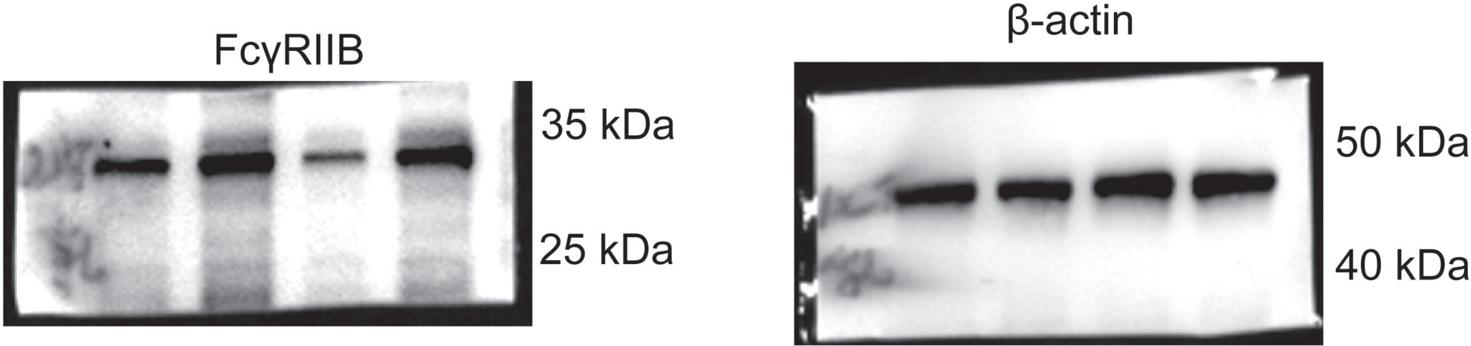
FcγRIIB
35 kDa
25 kDa
β-actin
50 kDa
40 kDa

Fig 8B

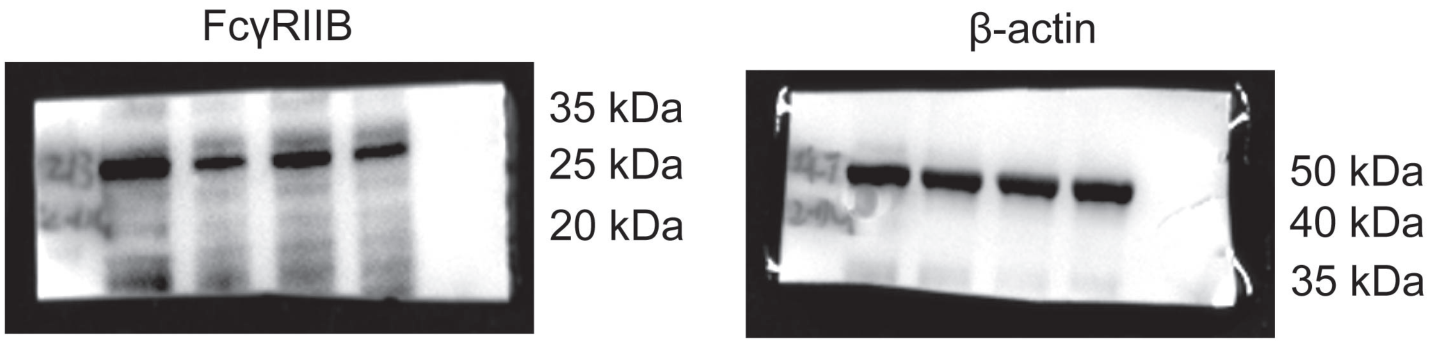

Fig 8D

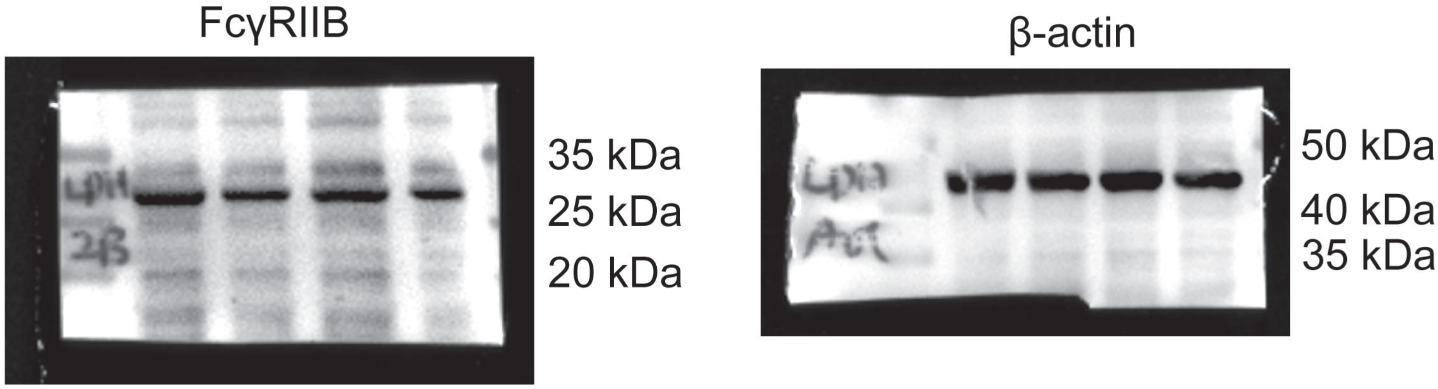

Fig 8H

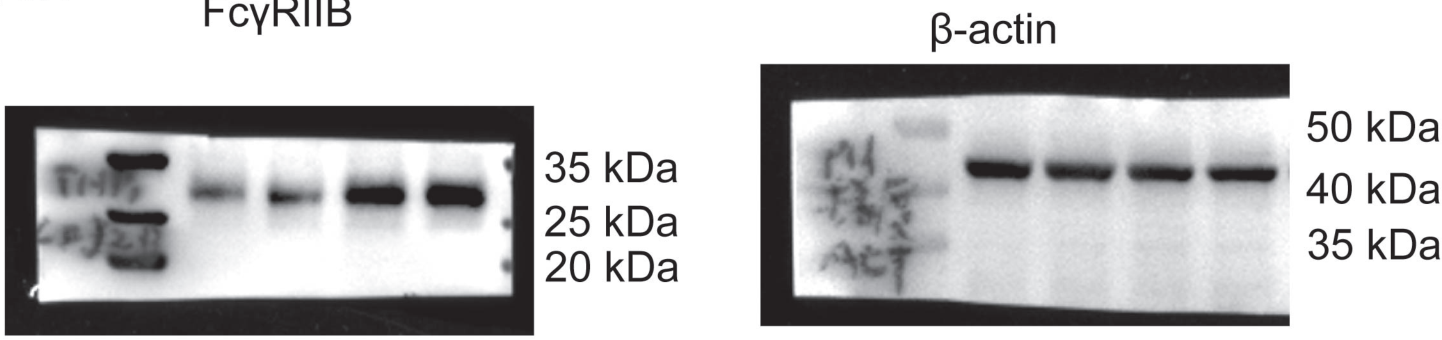

Fig 8J

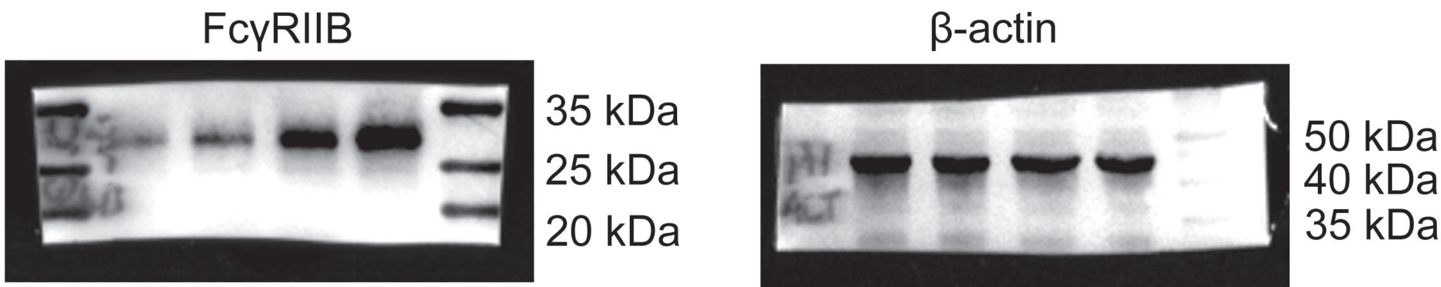

Fig 9G

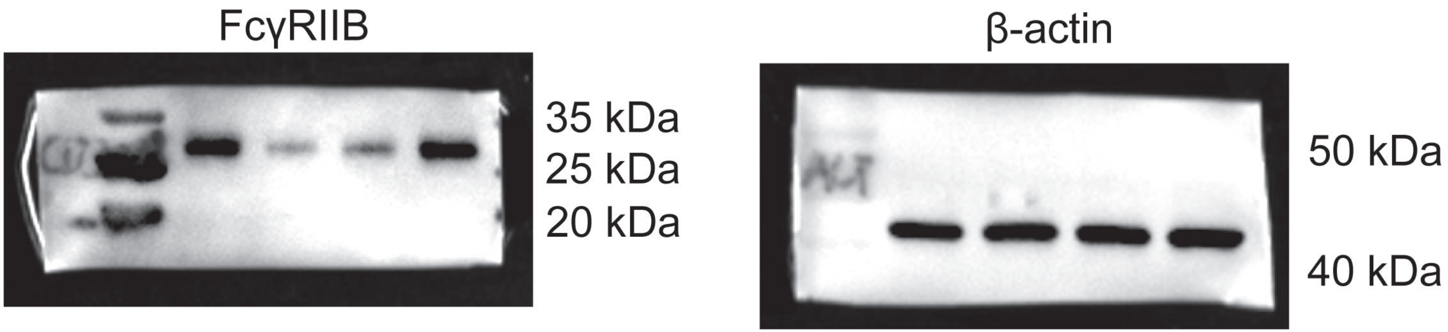

Supplementary Fig 6B

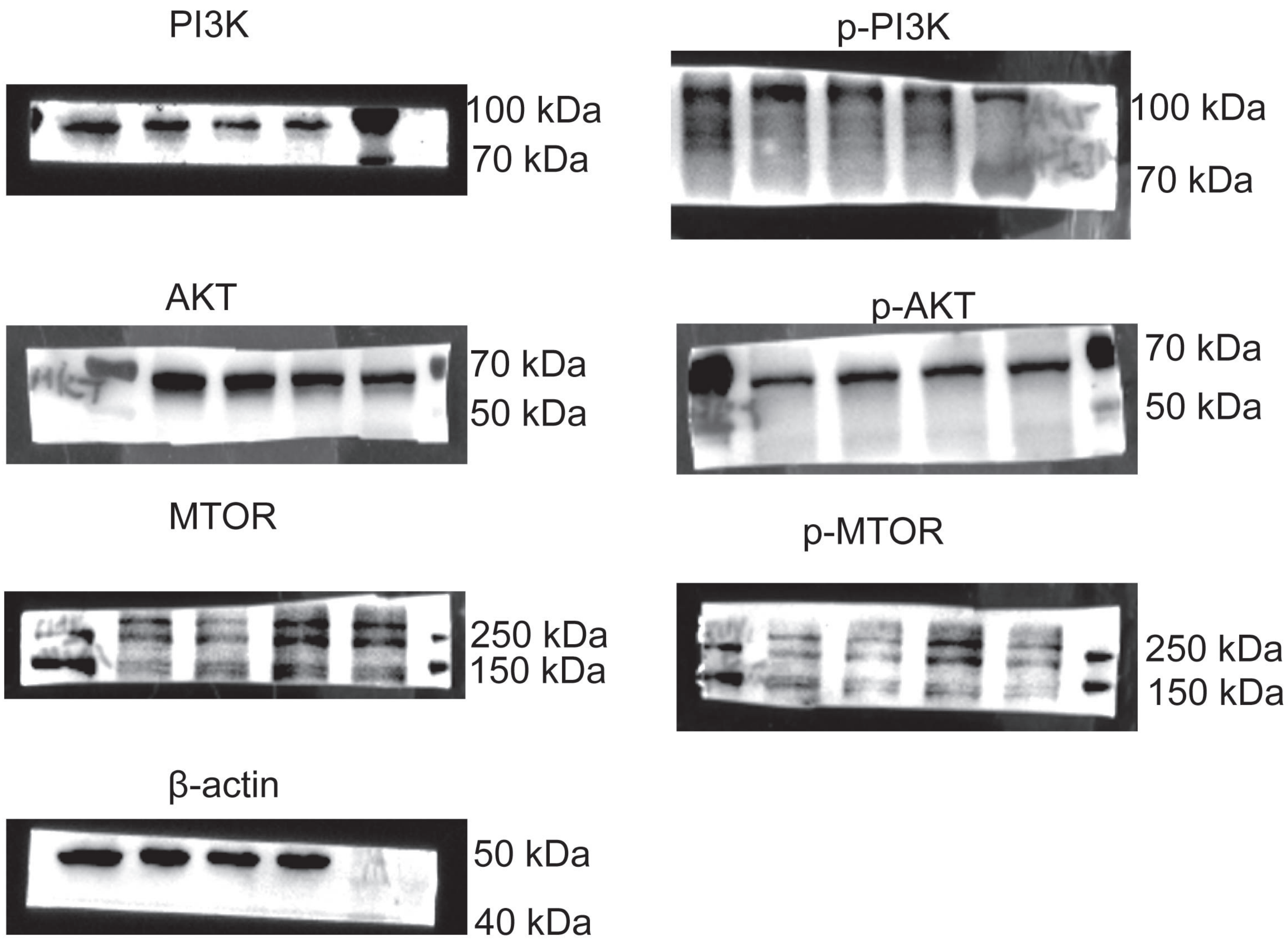

Supplementary Fig 6B

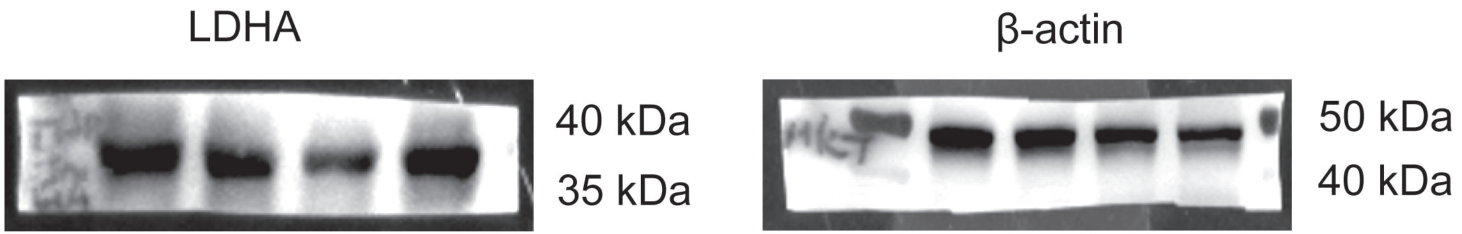

Supplementary Fig 6D

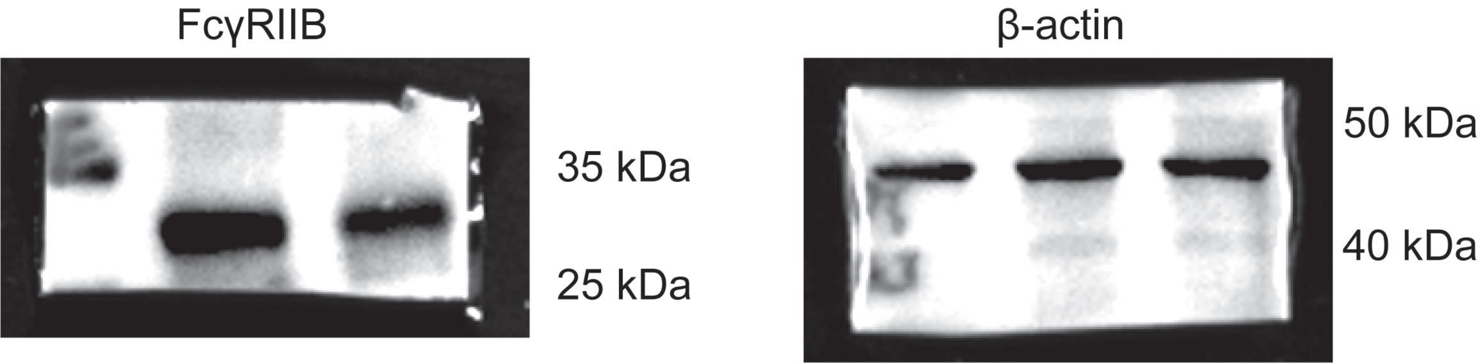

Supplement: Supplementary file 3 — Western blot [file 41419_2024_6875_MOESM3_ESM.pdf]
